# A Meta-Analysis Showing the Quantitative Evidence Base of Preemptive Pregabalin for Postoperative Pain from Cancer-Related Surgery

**DOI:** 10.3390/medicina59020280

**Published:** 2023-01-31

**Authors:** Qian Wang, Jing Dong, Xin Ye, Yi-Feng Ren

**Affiliations:** Hospital of Chengdu University of Traditional Chinese Medicine, Chengdu 610072, China

**Keywords:** preemptive, pregabalin, cancer-related surgery, meta-analysis

## Abstract

*Background and Objectives*: As an adjunct to postoperative multimodal analgesic regimens, pregabalin has been reported in reducing postoperative acute pain and opioid consumption. However, there is only a small amount of evidence for preemptive pregabalin in patients undergoing cancer-related surgery. This systematic review was conducted to integrate high-quality evidence to evaluate the preemptive analgesic effects of pregabalin in cancer-related surgery. *Materials and Methods*: Seven electronic databases were searched in a combination of subject terms and free words. Efficacy and safety of preemptive pregabalin on postoperative pain for cancer-related surgery were evaluated by assessing resting and dynamic pain scores postoperatively, cumulative morphine equivalent consumption, time to first analgesic request, hemodynamic parameters, and the safety indicators. *Results*: Thirteen trials were incorporated for quantitative synthesis. The pooled results showed administration of pregabalin preoperatively is clinically significant for improving resting (weighted mean difference (WMD), −1.53 cm; 95% CI, −2.30 to −0.77) and dynamic (WMD, −1.16 cm; 95% CI, −2.22 to −0.11) pain severity scores at 2 h postoperatively and prolonging time to first analgesic request (WMD, 2.28 h; 95% CI, 0.79 to 3.77) in cancer-related surgery. Preemptive pregabalin was also statistically effective in some other pain indicators but would increase the risk of pregabalin-related side effects after surgery. *Conclusions*: Our findings do not support the administration of pregabalin in doses larger than 300 mg when put in cancer-related surgery. Taken together, more high-quality research particularly focused on the optimal dosages and timing of pregabalin in cancer-related surgery is needed in the future to establish stronger evidence for therapeutic effects.

## 1. Introduction

Surgical resection of primary tumors is a building block for cancer treatment that provides tremendous benefits to survival [1]. However, postoperative acute pain caused by a wide range of surgical incisions and tissue and neural damage is a challenging issue in patients undergoing cancer-related surgery. Early, acute, and persistent postoperative pain is considered to be the most common chief complaint in patients presenting for cancer surgery and leads to an increased risk of chronic pain and a reduction in quality of life [2,3,4].

Opioids, such as morphine, are the most widely used prescription analgesics for acute moderate to severe postoperative pain in cancer-related surgery [5,6]. However, when high doses of opioids are used, in addition to the risks of respiratory depression, tolerance, nausea, and vomiting (PONV) [7], they can also increase the risks of metastasis and recurrence [8]. Opioid has potential tumorigenic effects by μ-signaling pathways and sympathetic nervous system (SNS) and hypothalamic-pituitary-adrenal (HPA) axis in vitro and in vivo tests. However, there is conflicting evidence from current clinical studies [9,10,11]. Regardless, it is essential to use opioids with a degree of caution and seek alternative measures, such as multimodal and preemptive analgesia protocols, to improve acute pain management in cancer-related surgery.

Postoperative pain arises through multiple physiological and pathological mechanisms, including nociceptive inputs resulting from incision and peripheral and central sensitization via nerve growth factor, cytokines, and α-amino-3-hydroxy-5-methyl-4-isoxazolepropionic acid receptor [12]. On the other hand, surgical insult and nerve damage caused by surgical removal is the basis of postoperative acute pain [13]. Thus, anticonvulsants characterized as anti-hyperalgesic, such as the gabapentin class of drugs (gabapentin and pregabalin), were introduced as an adjunct to perioperative multimodal analgesic regimens [14]. Compared with gabapentin, pregabalin has better pharmacokinetic characteristics, fast absorption, high bioavailability, and low inter-subject variability [15]. These added advantages in analgesia make pregabalin a more attractive option. In addition, pregabalin is thought to be more effective in preventing neuropathic components of surgical acute nociceptive pain, producing more opioid retention effects, and improving perioperative anxiety [16].

Previous studies and systematic reviews have reported the effect of pregabalin as a perioperative adjuvant analgesic in reducing postoperative acute pain and adverse effects by reducing the opioid dose and pain scores [17,18,19]. However, there is only a small amount of evidence for preemptive pregabalin in patients undergoing cancer-related surgery. Given the above information, it was decided to integrate high-quality evidence to evaluate and analyze the preemptive analgesic effects and safety of pregabalin in cancer-related surgery.

## 2. Materials and Methods

### 2.1. Search Strategy

Our meta-analysis was prepared and reported strictly in accordance with the Preferred Reporting Items for Systematic Reviews and Meta-Analyses (PRISMA), Assessing the Methodological Quality of Systematic Reviews (AMSTAR), and the Cochrane Collaboration [20,21]. Analyses were conducted according to a predesigned protocol that had been registered with the International Prospective Register of Systematic Reviews (PROSPERO) (CRD42021251250, https://www.crd.york.ac.uk/prospero/display_record.php?RecordID=251250, accessed on 24 May 2021). We carried out a systematic search strategy from the following seven electronic databases: PubMed, Embase, Web of Science, the Cochrane Library, Google Scholar, China National Knowledge Infrastructure (CNKI), and the Wan-Fang database. In addition, two trial registries were searched in search of suitable studies (https://www.ClinicalTrials.gov and the International Clinical Trial Registry Platform). The last retrieval date was 8 May 2022.

An information specialist (QLW) with extensive experience in systematic reviews and meta-analysis assisted with the search process. To avoid omission, subject terms and free words were included as part of the structured search strategy such as ‘Pregabalin’, ‘Neoplasms’, ‘General Surgery’, ‘Surgical Procedures, Operative’, and ‘random’. It was not restricted in terms of language, publication year, journal, or geographical location. A detailed description of the retrieval strategy is provided in the Supplementary Materials (Methods S1–S5).

### 2.2. Eligibility Criteria and Study Selection

Inclusion criteria:

Participants: Patients who underwent cancer-related surgery were included. In particular, patients who underwent radiofrequency ablation were excluded in order to reduce potential confounding variables. Study subjects were of both sexes, from the age of 18–80 years.

Interventions: Pregabalin was administered preoperatively.

Comparator: Control interventions consisted of placebos or no treatment.

Outcomes: We included studies that reported at least one of the following outcomes: pain-related outcomes, hemodynamic parameters, and safety indicators.

Study design: Only articles reporting randomized controlled trials (RCTs) were included in our review.

Exclusion criteria: (1) Studies in which pregabalin was outside the surgical setting or not used for cancer-related surgery. (2) Studies that focused on the intraoperative or postoperative use of pregabalin. (3) Studies presented as retrospective observational studies, case reports, case series, review articles, or letters to the editor.

Two reviewers independently examined the titles and abstracts (QW and JD). After that, the full texts of potentially eligible articles were screened and assessed by two reviewers individually (QW and JD). All disagreements between reviewers were resolved by consensus or adjudication with a third arbitrator (XY).

### 2.3. Data Extraction

Two reviewers extracted demographic information and postoperative outcomes with a standardized data extraction form in Excel (Microsoft Excel 2019, Microsoft, Redmond, USA) (QW and JD). The extracted data included the following: the name of the first author, year of publication, types of cancer, surgical approach, anesthesia methods, sample size, intervention details, comparison group, age, number of interventions, methods of postoperative rescue analgesia, and outcomes. The final tables were reviewed by all authors. Data that were provided only in graphs were extracted with the software GetData Graph Digitizer version 2.26 (http://getdata-graph-digitizer.com/, accessed on 14 March 2022). In the case of missing information, we consulted the corresponding authors. We attempted to contact the authors of Salah 2018 for unclear baseline information, but no response was obtained [22].

### 2.4. Assessment of Methodological Quality and Risk of Bias

Two reviewers independently evaluated the risk of bias assessment of the included studies with the criteria outlined in the modified Cochrane Collaboration tool (ROB2) (QW and JD) [23]. Studies were categorized as having a high risk of bias, low risk of bias, or some concerns in the following six domains: (i) randomization process, (ii) deviations from the intended interventions, (iii) missing outcome data, (iv) measurement of the outcome, (v) selection of the reported result, and (vi) overall bias. Full details were shown in the Supplemental Material (Table S1).

Furthermore, quality assessment was executed according to the Grades of Recommendation, Assessment, Development, and Evaluation Profiler software (GRADEpro, version 3.6.1, McMaster University, Hamilton, ON, Canada). Evidence strength levels were assessed as high (⊕⊕⊕⊕), moderate (⊕⊕⊕◯), low (⊕⊕◯◯) or very low (⊕◯◯◯) based on the risk of bias, imprecision, heterogeneity, indirectness, and publication bias [24,25,26]. Specific details could be found in the Supplemental Material (Table S2).

Two reviewers independently conducted the quality assessments of the included RCTs (QW and JD). In the case of discrepancies between two reviewers, an independent reviewer resolved them (YFR).

### 2.5. Primary and Secondary Outcomes

The co-primary outcomes included the following: (1) resting pain scores postoperatively at 24 h (cm); (2) dynamic pain scores postoperatively at 24 h (cm). As a result of its frequent reporting, we chose this time point [22,27,28,29,30,31,32,33,34,35,36].

The secondary outcomes included the following: (1) resting pain scores postoperatively at other time points (cm); (2) dynamic pain scores postoperatively at other time points (cm); (3) cumulative morphine equivalent consumption within 12, 24, and 48 h postoperatively (mg); (4) time to first analgesic request (hours); and (5) hemodynamic parameters included heart rate (beat/min), systolic blood pressure (SBP) (mm/Hg) as well as diastolic blood pressure (DBP) (mm/Hg) at 2, 6, 12, and 24 h. We chose hemodynamic parameters as the secondary outcomes for 2 reasons. First, the release of cortisol and catecholamines mediated by pain results in heart rate and blood pressure increase, hence hemodynamic parameters were chosen as indirect physiological changes of pain [37]. Second, the time points selected were based on its frequent reporting in included studies.

The safety indicators included: (1) pregabalin-related side effects (such as dizziness, visual disturbance, pruritus, headache, and sedation); (2) opioid-related side effects (such as nausea and vomiting).

### 2.6. Analysis of Outcome Data

We converted all pain scores to a straight line of 0–10 cm in length (score range: 0 [no pain] to 10 [maximum pain]). In addition, the opioid consumption data were transformed into intravenous morphine equivalents (intravenous fentanyl 0.1 mg = intravenous tramadol 100 mg = intravenous morphine 10 mg) [38,39].

### 2.7. Statistical Analysis

The mean ± standard deviation (SD) is used for continuous variables (such as pain scores, cumulative morphine equivalent consumption, time to first analgesic request, hemodynamic parameters, and sedation score). All median, range, and/or interquartile ranges were converted to mean and SD according to instructions from Luo et al. [40]. As for categorical variables, we described them using counts and proportions. Pain scores, cumulative morphine equivalent consumption, time to first analgesic request, heart rate, SBP, DBP, and sedation score were represented as mean ± SD. The occurrence of dizziness, visual disturbance, pruritus, headache, and PONV were represented as counts and proportions.

### 2.8. Meta-Analysis

Meta-analysis was performed with Review Manager software (version 5.4, The Cochrane Collaboration, UK) and Stata software V. 16.0 (StataCorp, College Station, TX, USA). We used the Mantel–Haenszel method to conduct the dichotomous variables, and estimates for continuous variables were calculated using the inverse variance method [41]. We compiled the available data from included RCTs and the weighted mean difference (WMD) with 97.5% confidence intervals (CI) was calculated for co-primary outcomes to adjust for multiple comparisons [42]. Differences are considered significant for *p* < 0.025. Additionally, for dichotomous outcomes and other continuous data, we calculated the risk ratio (RR) with 95% CI or the WMD with 95% CI (two-sided *p* < 0.05 was considered significant).

We performed quantitative analysis when two or more RCTs reported similar measurable parameters. In our qualitative analysis, study characteristics and postoperative outcomes were described.

### 2.9. Interpretation of Outcome Results

We estimated differences between pregabalin and the control group for pain scores and intravenous morphine equivalent consumption using the minimal clinically important difference (MCID). According to previous literature, a decrease of 1.1 cm and 10 mg, as well as a prolonged period of 1 h was considered as MCIDs for the pain scores, intravenous morphine equivalent consumption, and time to first analgesic request [43,44,45].

### 2.10. Heterogeneity, Subgroup, and Sensitivity Analyses

We assessed the statistical heterogeneity in accordance with the Cochrane Handbook for Systematic Reviews of Interventions. The statistical heterogeneity was quantified by the Chi-squared (Chi^2^) test and I-square (I^2^) and was classified as low (I^2^ of 0% to 40%), moderate (I^2^ of 30% to 60%), substantial (I^2^ of 50% to 90%), or considerable (I^2^ of 75% to 100%) [21]. We chose the appropriate statistical method according to the value of I^2^: A fixed-effects model was used when heterogeneity was low (I^2^ < 50%), and a random-effects model was used when heterogeneity was high (I^2^ ≥ 50%). Meta-regression analysis with a random-effects model was conducted for co-primary outcomes to explore sources of heterogeneity. We used the coefficient of determination (R^2^) (range, 0 to 1) to account for the relationship between the covariates and heterogeneity. For subgroup analyses, it was performed only when a covariate was significant in the meta-regression. Predefined covariates were as follows: (1) Types of cancer (breast cancer vs. no breast cancer); (2) The dose of pregabalin (low dose (<300 mg) vs. high dose (≥300 mg)); (3) Types of surgery (radical surgical resection vs. non-radical surgical resection); (4) Methods of postoperative rescue analgesia (PCA (Patient controlled analgesia) vs. IV (Intravenous injection) vs. IM (Intramuscular injection)). Post hoc subgroup analyses were conducted as follows: (1) surgical site (head vs. thorax vs. abdomen); (2) postoperative multimodal analgesia (yes vs. no). Additional subgroup analyses about the dose of pregabalin were performed for safety outcomes to explore associations between dose and side effects.

To assess the robustness of our findings, sensitivity analyses were conducted only for co-primary outcomes by excluding trials reported by Mansor et al. [32] in which patients underwent general anesthesia plus local anesthesia.

### 2.11. Assessment of Publication Biases

Egger’s regression test was used to investigate publication bias in all outcomes according to Stata software V. 16.0 (StataCorp, College Station, TX, USA). Publication bias was considered significant if *p* < 0.05 [46].

## 3. Results

**Figure 1:**
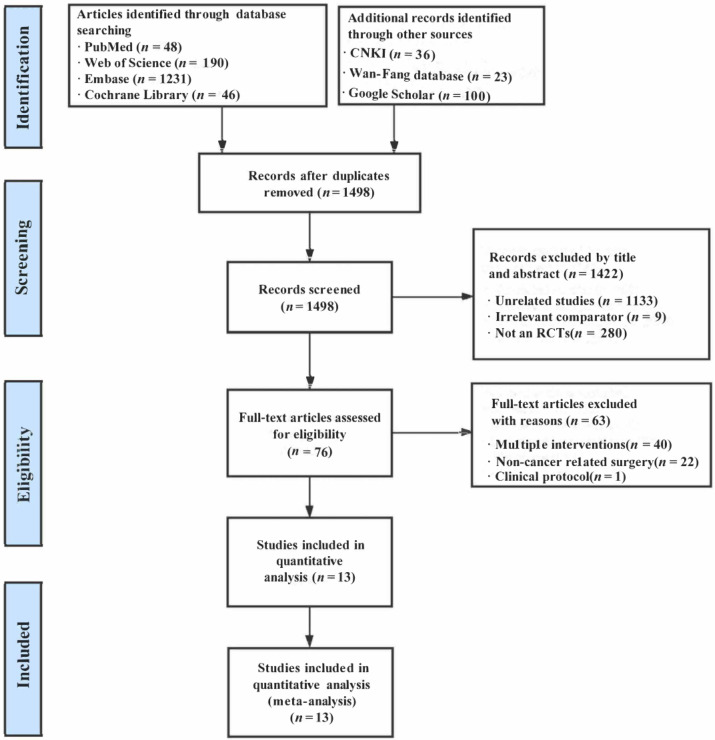
The flowchart of study selection.

The preliminary literature search identified a total of 1674 records. After removing 176 duplicates, 1498 citations were evaluated based on titles and abstracts. Among the 1498 citations, we excluded 1422 citations because of irrelevant studies (*n* = 1133), unrelated comparators (*n* = 9), or they were not designated as RCTs (*n* = 280). A total of 76 articles were eligible for full-text review, and 63 articles were excluded from analysis due to irrelevant intervention (*n* = 40), non-cancer-related surgery (*n* = 22), and the clinical protocol (*n* = 1). Finally, 13 trials (from 30 to 111 patients) were incorporated for quantitative synthesis [22,27,28,29,30,31,32,33,34,35,36,47,48]. The PRISMA flow diagram of this review was illustrated in Figure 1.

### 3.1. Characteristics of Included Studies

The outcomes of interest and trial characteristics were summarized in Table 1. The cancer type was breast cancer in eight out of the 13 included trials [27,29,31,32,35,36,47,48]. The other five trials that were not breast cancer included bladder cancer, supratentorial tumor, gynecological malignancies, lung cancer, and pleural cancer [22,28,30,33,34]. Twelve trials were carried out under general anesthesia [22,27,28,29,30,31,33,34,35,36,47,48] and one trial was performed under general plus local anesthesia which was incisional infiltration consisting of levobupivacaine [32]. The postoperative rescue analgesic was morphine in all studies [22,27,28,29,31,33,34,35,36,47,48], except for two studies that used fentanyl [30] or tramadol [32], respectively. PCA was used in eight studies [22,27,28,29,30,31,33,48], IV was used in two studies [32,47], and IM was used in three studies [34,35,36]. Eight studies enrolled patients who underwent radical surgery [27,28,29,31,33,35,36,47], whereas three studies enrolled those who underwent non-radical surgery [30,32,48] and the remaining two studies did not specify the type of surgery [22,34]. The dose of pregabalin ranged from 75 mg to 600 mg. Multiple doses of pregabalin were administered only in one trial [28] while single-dose treatment preoperatively was administered in other trials. The control group received a placebo in all studies except one trial that had no intervention [28].

### 3.2. Risk of Bias

**Figure 2:**
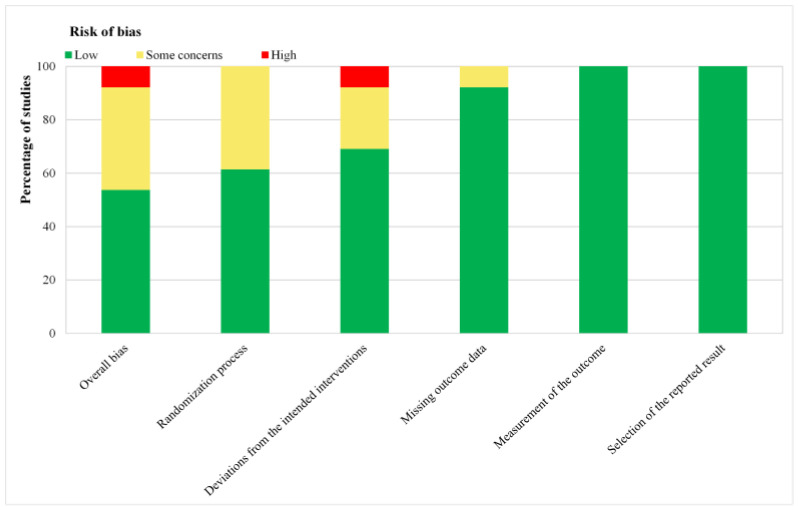
Risk of bias graph: review authors’ judgments about each risk of bias item presented as percentages across all included studies. Green for low risk of bias, yellow for unclear risk of bias, and red for high risk of bias.

According to the tool of Cochrane Collaboration, seven trials demonstrated low [27,29,31,32,33,47,48] and five trials [22,30,34,35,36] demonstrated some concerns, respectively, and one trial demonstrated high bias risk (Figure S1 and Figure 2) [28]. The results and details of the risk of bias assessments were presented in Table S1.

### 3.3. Primary Indicators

#### 3.3.1. Resting Pain Scores at 24 h Postoperatively (cm)

Nine studies evaluated resting pain scores at 24 h after surgery (*n* = 580) (Table 2) [22,27,28,29,31,32,33,34,35]. The pregabalin group was statistically different for this result compared to the control group but did not reach the clinical threshold of 1.1 cm (WMD, −0.45 cm; 97.5% CI, −0.68 to −0.21; *p <* 0.001; I^2^ = 63.32%) (Table 2). Meta-regression analyses showed that types of cancer (R^2^ = 0, *p* = 0.712), the dose of pregabalin (R^2^ = 0, *p* = 0.800), types of surgery (R^2^ = 0, *p* = 0.654), methods of postoperative rescue analgesia (R^2^ = 0.23, *p* = 0.056), surgical site (R^2^ = 0, *p* = 0.717), and postoperative multimodal analgesia (R^2^ = 0.22, *p* = 0.202) were not the possible sources of heterogeneity for resting pain scores at 24 h postoperatively (Table S3). No subgroup analysis was performed because of the meaningless result of meta-regression. The results of sensitivity analyses were not significantly different from those of preliminary analyses, which indicated that the results were robust and reliable (Table S4B).

According to the GRADE criteria, the quality of evidence was rated as ‘low’ for this outcome because of heterogeneity and risk of bias (Table S2). There was no publication bias in this outcome (*p* = 0.87).

#### 3.3.2. Dynamic Pain Scores at 24 h Postoperatively (cm)

A total of seven studies assessed dynamic pain scores at 24 h after surgery (*n* = 490) (Table 2) [27,28,29,31,32,34,36]. There was no significant difference in this outcome (*p* = 0.19) between the pregabalin group and the control group. Meta-regression analyses showed that methods of postoperative rescue analgesia (R^2^ = 0.54, *p* = 0.047) were the possible sources of heterogeneity for this result (Table S3). Although, types of cancer (R^2^ = 0, *p* = 0.847), the dose of pregabalin (R^2^ = 0, *p* = 0.587), types of surgery (R^2^ = 0, *p* = 0.890), surgical site (R^2^ = 0, *p* = 0.846), and postoperative multimodal analgesia (R^2^ = 0.437, *p* = 0.290) were not the possible sources of heterogeneity for this result. Subgroup analyses showed that pregabalin had a significant effect on this outcome in the IM cohort (WMD, −0.87 cm; 97.5% CI, −1.42 to −0.33; *p* = 0.01; I^2^ = 86.11%), whereas there was no significant effect in the PCA (*p* = 0.39) and IV cohorts (*p* = N/A) compared to the control group (Table S4A). Pre-planned sensitivity analyses indicated no statistically significant differences between the two groups (*p* = 0.11) (Table S4B).

The quality of evidence was ‘low’ for the outcome as a result of heterogeneity and risk of bias (Table S2). Egger’s regression test indicated no publication bias (*p* = 0.12).

### 3.4. Secondary Indicators

#### 3.4.1. Resting Pain Scores at Individual Time Points Postoperatively (cm)

**Figure 3:**
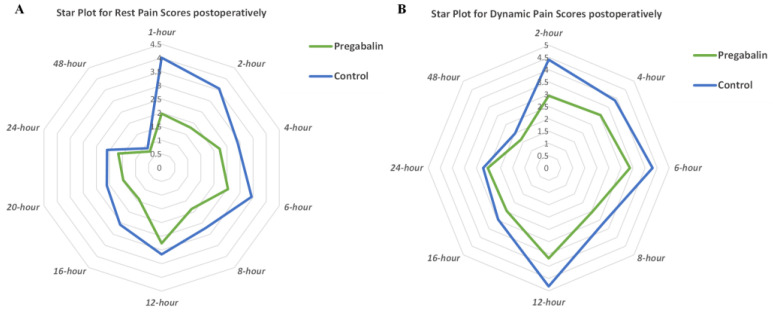
(**A**) Star plot for weighted mean of resting pain scores within 48 h after cancer-related surgery at ten time points in pregabalin and control groups; (**B**) star plot for weighted mean of dynamic pain scores within 48 h after cancer-related surgery at eight time points in pregabalin and control groups.

Resting pain scores were analyzed at a period of 1 h (two studies, *n* = 165) [34,35], 2 h (seven studies, *n* = 510) [29,31,32,33,34,35,47], 4 h (seven studies, *n* = 465) [22,28,29,31,32,33,47], 6 h (seven studies, *n* = 429) [27,31,32,33,34,35,47], 8 h (four studies, *n* = 311) [22,28,29,47], 12 h (eight studies, *n* = 500) [22,27,28,31,33,34,35,47], 16 h (three studies, *n* = 246) [28,29,35], 20 h (two studies, *n* = 135) [28,35], and 48 h (two studies, *n* = 90) (Table 2) [27,28]. The pooled results varied over time, and integrative analyses showed that preoperative oral pregabalin significantly reduced postoperative resting pain scores at 2 h (WMD, −1.53 cm; 95% CI, −2.30 to −0.77; *p* < 0.001; I^2^ = 97%), 4 h (WMD, −0.53 cm; 95% CI, −0.98 to −0.08; *p* = 0.02; I^2^ = 92%), 6 h (WMD, −0.87 cm; 95% CI, −1.58 to −0.16; *p* = 0.02; I^2^ = 95%), 8 h (WMD, −0.64 cm; 95% CI, −0.96 to −0.32; *p* < 0.001; I^2^ = 57%), 12 h (WMD, −0.59 cm; 95% CI, −1.06 to −0.12; *p* = 0.01; I^2^ = 88%), 16 h (WMD, −1.07 cm; 95% CI, −1.88 to −0.25; *p* = 0.01; I^2^ = 91%), and 20 h (WMD, −0.61 cm; 95% CI, −1.18 to −0.05; *p* = 0.03; I^2^ = 78%) relative to the control group (Table 2). The star plot for the weighted mean of resting pain scores within 48 h after cancer-related surgery at ten time points in pregabalin and control groups was shown in Figure 3A. The difference for resting pain scores at 2 h postoperatively met the clinical threshold of MCID (1.1 cm), whereas other results did not. Moreover, analyses showed no significant difference in resting pain scores at 1 h (*p* = 0.14) and 48 h (*p* = 0.36) (Table 2).

The quality of evidence for these outcomes was evaluated as ‘low’ or ‘moderate’ owing to the risk of bias, heterogeneity, and inaccurate or publication bias (Table S2). Analyses showed that these results did not exhibit publication bias (all *p* > 0.05) except resting pain scores at 4 h (*p* = 0.03).

#### 3.4.2. Dynamic Pain Scores at Individual Time Points Postoperatively (cm)

Dynamic pain scores were analyzed at a period of 2 h (five studies, *n* = 400) [29,31,32,34,36], 4 h (four studies, *n* = 280) [28,29,31,32], 6 h (five studies, *n* = 319) [27,31,32,34,36], 8 h (two studies, *n* = 171) [28,29], 12 h (five studies, *n* = 330) [27,28,31,34,36], 16 h (two studies, *n* = 171) [28,29], and 48 h (two studies, *n* = 90) (Table 2) [27,28]. There was variation in dynamic pain scores over time, and meta-analysis showed that preoperative oral pregabalin significantly reduced dynamic pain scores at 2 h (WMD, −1.16 cm; 95% CI, −2.22 to −0.11; *p* = 0.03; I^2^ = 98%), 4 h (WMD, −0.53 cm; 95% CI, −0.97 to −0.10; *p* = 0.02; I^2^ = 85%), 6 h (WMD, −1.03 cm; 95% CI, −1.83 to −0.23; *p* = 0.01; I^2^ = 95%), and 12 h (WMD, −0.85 cm; 95% CI, −1.49 to −0.21; *p* = 0.01; I^2^ = 90%) relative to the control group. The star plot for the weighted mean of dynamic pain scores within 48 h after cancer-related surgery at eight time points in pregabalin and control groups was shown in Figure 3B. According to the MCID (1.1 cm), differences were clinically meaningful for dynamic pain scores at 2 h, whereas other results were not. Moreover, analyses showed no significant difference in dynamic pain scores at 8 h (*p* = 0.10), 16 h (*p* = 0.07), and 48 h (*p* = 0.47) compared with the control group (Table 2).

Considering the high risk of bias or high heterogeneity, the quality of evidence for those results was evaluated as ‘low’ or ‘moderate’ (Table S2). Analyses showed that those outcomes had no publication bias (all *p* > 0.05).

### 3.5. Cumulative 12 to 48 h Morphine Equivalent Consumption (mg)

Some studies reported cumulative morphine equivalent consumption within 12 h (two studies, *n* = 110) [27,47], 24 h (10 studies, *n* = 646) [22,27,28,29,30,31,33,35,36,48], and 48 h (two studies, *n* = 90) (Table 2) [27,28]. The findings showed no statistically significant difference between pregabalin groups and control groups for morphine equivalent consumption within 12 h (*p* = 0.49) and 48 h (*p* = 0.26), but a statistically significant difference for it within 24 h (WMD, −7.45 mg; 95% CI, −9.30 to −5.60; *p* < 0.001; I^2^ = 96%) (Table 2).

The quality of evidence of these outcomes was ‘very low’ or ‘moderate’ due to the risk of bias or high heterogeneity (Table S2). Egger’s regression test indicated the absence of publication bias for morphine consumption within 12 h (*p* = 0.38), but cumulative morphine equivalent consumption within 24 h (*p* < 0.001) and 48 h (*p* = 0.007) exhibited a risk of publication bias.

### 3.6. Time to First Analgesic Request (Hours)

Four studies reported the time to first analgesic request (*n* = 255) (Table 2) [28,33,34,48]. Administration of pregabalin significantly prolonged the time-to-first analgesic request compared with the control group (WMD, 2.28 h; 95% CI, 0.79 to 3.77, *p* = 0.003, I^2^ = 100%) (Table 2). The quality of this outcome was ‘very low’ owing to inconsistency, imprecision, and risk of bias (Table S2). Analyses showed there was significant publication bias for the result (*p* < 0.001).

### 3.7. Hemodynamic Parameters

#### 3.7.1. Heart Rate at 2, 6, 12, and 24 h (beat/min)

Two studies (*n* = 135) reported heart rates at 2, 6, 12, and 24 h (Table 2) [33,34]. The meta-analyses showed that the effect of pregabalin on heart rate was not statistically significantly different from the effect of placebo at 2 h (*p* = 0.26), 6 h (*p* = 0.33), 12 h (*p* = 0.11), and 24 h (*p* = 0.26) (Table 2). The quality of evidence for these outcomes was ‘high’ (Table S2). These outcomes showed that there was no significant publication bias (all *p* > 0.05).

#### 3.7.2. SBP at 2, 6, 12, and 24 h (beat/min) (mm/Hg)

SBP was reported by two studies (*n* = 135) (Table 2) [33,34]. The merged results revealed no statistically significant difference between pregabalin and control groups in mean SBP at 2 h (*p* = 0.10), 6 h (*p* = 0.44), 12 h (*p* = 0.47), and 24 h (*p* = 0.92) (Table 2). The GRADE of evidence for SBP at 2 and 12 h was ‘moderate’ owing to inconsistency, whereas SBP at 6 and 24 h were ‘high’ (Table S2). No publication bias was detected for those results (all *p* > 0.05).

#### 3.7.3. DBP at 2, 6, 12, and 24 h (beat/min) (mm/Hg)

Two studies reported DBP (*n* = 135) (Table 2) [33,34]. There was an insignificant difference between pregabalin and control groups in mean DBP at 2 h (*p* = 0.95), 6 h (*p* = 0.16), 12 h (*p* = 0.71), and 24 h (*p* = 0.55) (Table 2). The quality of evidence for DBP at 2 and 6 h was represented as ‘high’, whereas it was ‘moderate’ at 12 and 24 h due to inconsistency (Table S2). It was suggested that publication bias was not present (all *p* > 0.05).

### 3.8. The Safety Indicators

#### 3.8.1. Pregabalin-Related Side Effects

- Dizziness

Seven studies evaluated dizziness (*n* = 490) (Table 2) [28,29,31,32,33,34,35]. Pregabalin likely resulted in a certain increase in the incidence of dizziness (RR, 2.81; 95% CI, 1.75 to 4.53; *p* < 0.001; I^2^ = 0%) (Table 2). In post-hoc subgroup analyses, statistical significance was observed for dizziness postoperatively after using a low dose of pregabalin (RR, 2.20; 95% CI, 1.38 to 3.48; *p* < 0.001; I^2^ = 0%) and a high dose of pregabalin (RR, 9.25; 95% CI, 3.22 to 26.54; *p* < 0.001; I^2^ = 9%) (Table S4A). Clearly, the RR of the high-dose group was higher than that of the low-dose group. According to the GRADE approach, the quality of evidence for dizziness was rated as ‘moderate’ owing to the risk of bias (Table S2). The results indicated no publication bias (*p* = 0.28).

- Visual disturbance

Six studies reported visual disturbance (*n* = 415) (Table 2) [28,29,31,32,33,34]. Oral pregabalin increased the risk of visual disturbance (RR, 3.04; 95% CI, 1.37 to 6.73; *p* = 0.006; I^2^ = 0%) compared with the control groups (Table 2). Subgroup analyses indicated that the low (RR, 2.13; 95% CI, 1.02 to 4.47, *p* = 0.05; I^2^ = 0%) and high (RR, 7.25; 95% CI, 2.75 to 19.07; *p* < 0.001; I^2^ = 0%) dose of pregabalin could statistically increase the incidence of visual disturbance (Table S4A). Additionally, in the high-dose group, the RR was higher than in the low-dose group. The quality of evidence for the outcome was ‘moderate’ owing to the risk of bias (Table S2). There was no publication bias (*p* = 0.52).

- Pruritus

Four studies reported pruritus (*n* = 220) (Table 2) [28,30,33,48]. There was no significant difference in pruritus between the pregabalin and control groups (*p* = 0.05) (Table 2). Subgroup analyses of the dose of pregabalin suggested that there was no statistical significance in the two subgroups (both *p* > 0.05) (Table S4A). The quality of evidence for pruritus was ‘moderate’ owing to the risk of bias (Table S2). No publication bias was determined (*p* = 0.31).

- Headache

Four studies assessed headaches (*n* = 295) (Table 2) [29,32,33,34]. Pregabalin had no difference in the incidence of headaches compared to the control groups (*p* = 0.25) (Table 2). The results for the incidence of pruritus in the low dose of pregabalin subgroup indicated no statistical significance (*p* = 0.38), whereas the high dose of pregabalin subgroup indicated statistical significance (RR, 5.11; 95% CI, 1.70 to 15.36; *p* = 0.004; I^2^ = 0%) (Table S4A). The quality of evidence for this outcome was ‘high’ (Table S2). Results revealed no presence of publication bias (*p* = 0.13).

- Sedation score at 12 h

Three studies evaluated sedation scores at 12 h (*n* = 230) (Table 2) [22,34,47]. The use of pregabalin likely significantly increased the sedation score at 12 h compared with the control groups (WMD, 0.35; 95% CI, 0.15, 0.55; *p* < 0.001; I^2^ = N/A) (Table 2). The results for this outcome on the low dose of pregabalin subgroup indicated statistical significance (WMD, 0.35; 95% CI, 0.15, 0.55; *p* < 0.001; I^2^ = N/A) (Table S4A). The quality of evidence for the sedation score at 12 h was ‘high’ (Table S2). Publication bias is inconclusive due to less data (*p* = N/A).

- Sedation score at 24 h

Three studies evaluated this outcome (*n* = 261) (Table 2) [22,29,34]. Pregabalin likely resulted in a significant increase in sedation score at 24 h (WMD, 0.50; 95% CI, 0.15 to 0.86; *p* = 0.006; I^2^ = 57%) (Table 2). The results for this result on the low dose of pregabalin subgroup indicated no statistical significance (*p* = 0.23), whereas the high dose of pregabalin subgroup indicated statistical significance (WMD, 0.76; 95% CI, 0.55 to 0.88; *p* < 0.001; I^2^ = 0%) (Table S4A). The quality of evidence for the outcome was ‘moderate’ owing to inconsistency (Table S2). Publication bias has not been proven for less data (*p* = N/A).

#### 3.8.2. Opioid-Related Side Effects

- PONV

PONV was reported in 10 studies (*n* = 670) (Table 2) [22,28,29,30,32,33,34,47,48]. The difference in the incidence of PONV was statistically significant between the pregabalin group and the control group (RR, 0.59; 95% CI, 0.39 to 0.87; *p* = 0.008; I^2^ = 60%) (Table 2). A significant statistical effect was observed for PONV postoperatively in a low dose of pregabalin (RR, 0.70; 95% CI, 0.55 to 0.90, *p* = 0.005; I^2^ = 24%) and a high dose of pregabalin (RR, 0.32; 95% CI, 0.19 to 0.52; *p* < 0.001; I^2^ = 0%) (Table S4A). The quality of evidence for POVN was ‘low’ due to the risk of bias and heterogeneity (Table S2), and the publication bias was not significant (*p* = 0.11). This section may be divided into subheadings. It should provide a concise and precise description of the experimental results, their interpretation, as well as the experimental conclusions that can be drawn.

## 4. Discussion

Thirteen RCTs (865 patients) were systematically reviewed and the role of preemptive pregabalin on cancer-related surgery was evaluated in this meta-analysis. The present study thoroughly summarized the current qualitative and quantitative evidence. The administration of pregabalin preoperatively is clinically significant for improving resting and dynamic pain severity scores at 2 h postoperatively (MCID: 1.1 cm) and prolonging time to first analgesic request (MCID: 1 h) in cancer-related surgery. Statistically, preemptive pregabalin was effective in resting and dynamic pain relief at most time points within 48 h postoperatively and opioid consumption within 24 h after surgery, but not clinically significant. On the other hand, there was no difference in opioid consumption at any other period and hemodynamic parameters between pregabalin and the control group. As can be seen from Table 2, non-significant results of hemodynamic parameters were in disagreement with results that pain scores at 2 h showed significant differences. One reasonable explanation is that although hemodynamic parameters could reflect the change of pain, they are also considered susceptible to other factors such as analgesic medications (including paracetamol, opioids, and non-steroidal anti-inflammatory drugs) [37,49,50,51]. Hence, analgesic medications such as opioids inducing hypotension may mask hemodynamic parameter changes induced by pain. Notably, compared with control groups, the risk of postoperative drug-associated adverse effects (including dizziness, visual disturbance, and sedation) was significantly increased with the use of pregabalin preoperatively. Due to the lack of sufficient data, severe adverse reactions like delirium could not be assessed. Additionally, the gabapentin class of drugs, especially at higher doses, may affect the metastatic progression of tumor cells through disruption of Ca2 + signaling in Mat-LyLu cells in prostate cancer [52,53]. Even if the available data are largely controversial and inadequate at present, the use of pregabalin warrants caution [53]. Consequently, the findings impaired the plausibility of using preemptive pregabalin in cancer-related surgery. Collectively, it may be prudent to seek additional evidence regarding the reasonableness of off-label usage of pregabalin, especially in situations that require balancing efficacy and potential risks of neurological complications and tumorigenic effects. In addition to drug analgesia, taking into account multimodal perioperative management principles and acute pain management requirements, basic anesthesia plus different analgesic techniques including regional anesthesia (like nerve block), local anesthesia (like incisional infiltration), and some new technologies (like sufentanil sublingual tablet system) could have potential in oncological surgery [54,55,56]. In this study, the results of a meta-regression analysis and subgroup analyses did not reveal any significant association between five covariates (including types of cancer, the dose of pregabalin, types of surgery, surgical site, and postoperative multimodal analgesia) and primary outcomes. Additionally, the exploratory subgroup analyses of the dose side effects indicated that a high dose of pregabalin is associated with more severe side effects (such as dizziness, visual disturbance, headache, and sedation). Another notable finding of this review is the apparent decrease in the incidence of PONV following low versus high doses of pregabalin. Based on these results, our current recommendation is not to use pregabalin in doses larger than 300 mg. Moreover, future research areas should focus on the assessment of the optimal dosages and timing of pregabalin in cancer-related surgery, to establish stronger evidence for therapeutic effects.

There has been considerable research on pregabalin in reducing pain intensity, morphine consumption, and side effects after surgery [17,19,57,58,59,60]. We had similar results with a recent review described by Chang et al. [61,62] for pain severity scores postoperatively in breast cancer surgery when pregabalin was administrated. However, an opposite conclusion was obtained from our study, which is most likely explained by different methods for the interpretation of results. Focusing on MCID allowed us to generate evidence with clinical merits. Comparatively, the findings of Rai et al. [62] pooled from two trials demonstrated that pregabalin did not reduce pain at 24 h undergoing breast cancer surgery, which was partly consistent with the results of our systematic review. Its insufficient sample size and low-quality evidence may lead to bias and limit external validity. One additional large meta-analysis also showed there was no clinically significant reduction in postoperative pain intensity with pregabalin in adult patients undergoing surgery [63].

Pharmacologically, the pregabalin binds potently to the alpha-2-delta subunit of voltage-sensitive calcium channels to reduce depolarization-induced calcium influx at nerve terminals and the release of several neurotransmitters (including glutamate and norepinephrine) which were induced by nerve injury related to cancer resection [27,28,64,65]. Thus, it is no wonder that pregabalin can reduce postoperative pain. From a pharmacokinetics perspective, pregabalin has a plasma half-life of 6.5 h and takes approximately 1 h to reach maximum concentration in circulation [66]. Moreover, preemptive analgesia-blocking afferent nerve fibers before pain stimulation alters peripheral and central nervous system processing of harmful stimuli, reduces nociceptive inputs to the central nervous system, and optimizes perioperative analgesia. This may explain why pregabalin only contributes to clinical improvement in the early stage of postoperative pain (2 h after surgery).

There were some advantages to the present study. First, we conducted an a priori register of the review at PROSPERO to minimize duplication of work and publication bias. Second, we conducted a comprehensive literature search, including a thorough search of trial registration platforms and the grey literature. Third, the clinical values of pain intensity and morphine consumption were defined as cut-points for important improvement using MCID. Fourth, corrections for multiple comparisons were conducted for co-primary outcomes and the confidence interval was set at 97.5% to reduce the possibility of a false positive association. Finally, an exploratory dose side effect analysis was performed in order to examine the risk of adverse effects at different dosages and presents some important findings.

There were some limitations to the study. First, the review included a small number of RCTs with small sample sizes which could lead to concerns about bias. Second, moderate-to-high heterogeneity was observed in some outcome measures and resulted in lowering evidence grades. This may be attributed to methods of postoperative rescue analgesia according to the subgroup analyses. Third, these results may not accurately reflect the true picture of the dose side effect due to the limited data on safety indicators. Fourth, we excluded the RCTs of radiofrequency ablation, which may impair the generalizability of our findings. Fifth, limited data is not sufficient to draw conclusions about chronic pain. Finally, we did not include the study from Lamsal et al. in the primary outcome because of unadjusted or compatible data and we were not able to obtain the data from the authors.

## 5. Conclusions

Pooled evidence suggests that preemptive pregabalin in cancer-related surgery could clinically significantly improve acute pain after surgery (only at 2 h) and time to first analgesic request. In addition, pregabalin provides a marginally significant reduction for resting and dynamic pain severity scores at most time points within 48 h postoperatively, and opioid consumption within 24 h after surgery. It is worth noting that the high dose of pregabalin is associated with more severe side effects. Our findings do not support the administration of pregabalin in doses larger than 300 mg when put in cancer-related surgery. Taken together, more high-quality research particularly focused on the optimal dosages and timing of pregabalin in cancer-related surgery is needed in the future to establish stronger evidence for therapeutic effects.

## Figures and Tables

**Table 1:** Characteristics of included studies.

Authors (Year)	Cancer Types	Surgical Site	Surgical Approach (Anesthesia Methods)	Groups (*N*): Treatment		Number of Interventions	Rescue Analgesia	Outcomes
				Intervention	Control			
Earsakul (2017) [27]	Breast cancer	Thorax	MRM or mastectomy with ALND (GA)	Pregabalin (16): 75 mg PO	Control (14): placebo PO	Single	Morphine PCA	(1) Resting and dynamic pain severity scores (2) Cumulative morphine equivalent consumption (3) Postoperative adverse effects and complications
Ghoneim et al. (2013) [28]	Bladder cancer	Abdomen	Radical cystectomy with urinary diversion (GA)	Pregabalin (30): 75 mg PO	Control (30): no intervention	Multiple	MorphinePCA + Paracetamol IV	(1) Resting and dynamic pain severity scores (2) Time to first rescue analgesia (3) Cumulative morphine equivalent consumption (4) Postoperative adverse effects and complications
Hetta et al. (2016) [29]	Breast cancer	Thorax	MRM (GA)	Pregabalin (28): 75 mg PO Pregabalin (27): 150 mg PO Pregabalin (26): 300 mg PO	Control (30): placebo PO	Single	Morphine PCA	(1) Resting and dynamic pain severity scores (2) Cumulative morphine equivalent consumption (3) Postoperative adverse effects and complications
Lamsal et al. (2019) [30]	Supratentorial tumor	Head	Supratentorial craniotomy (GA)	Pregabalin (18): 75 mg PO Pregabalin (19): 150 mg PO	Control (18): placebo PO	Single	Fentanyl PCA	(1) Resting pain severity scores (2) Cumulative fentanyl equivalent consumption (3) Postoperative adverse effects and complications
Mahran et al. (2015) [31]	Breast cancer	Thorax	MRM (GA)	Pregabalin (30): 150 mg PO	Control (30): placebo PO	Single	Morphine PCA	(1) Resting and dynamic pain severity scores (2) Cumulative morphine equivalent consumption (3) Postoperative adverse effects and complications
Mansor et al. (2015) [32]	Breast cancer	Thorax	Mastectomy (GA+LA)	Pregabalin (25): 150 mg PO	Control (24): placebo PO	Single	Tramadol IV + Etoricoxib and Paracetamol PO	(1) Resting and dynamic pain severity scores (2) Postoperative adverse effects and complications
Mohamed et al. (2016) [33]	Urinary bladder cancer	Abdomen	Radical cystectomy (GA)	Pregabalin (15): 300 mg PO Pregabalin (15): 600 mg PO	Control (15): placebo PO	Single	Morphine PCA	(1) Resting pain severity scores (2) Cumulative morphine equivalent consumption (3) Time to first rescue analgesia (4) Hemodynamic parameters (5) Postoperative adverse effects and complications
Patel et al. (2016) [34]	Gynecological malignancies	Abdomen	Abdominal hysterectomy and bilateral salphyngo—oophorectomy (GA)	Pregabalin (30): 150 mg PO Pregabalin (30): 300 mg PO	Control (30): placebo PO	Single	Morphine IM	(1) Resting and dynamic pain severity scores (2) Time to first rescue analgesia (3) Cumulative morphine equivalent consumption (4) Hemodynamic parameters (5) Postoperative adverse effects and complications
SK et al. (2016) [47]	Breast cancer	Thorax	MRM (GA)	Pregabalin (40): 150 mg PO	Control (40): placebo PO	Single	Morphine IV	(1) Resting pain severity scores (2) Cumulative morphine equivalent consumption (3) Postoperative adverse effects and complications
Pushkarna et al. (2022) [48]	Breast cancer	Thorax	BCCS (GA)	Pregabalin (30): 75 mg PO	Control (30): placebo PO	Single	Morphine PCA + Diclofenac IV	(1) Cumulative morphine equivalent consumption (2) Time to first rescue analgesia (3) Postoperative adverse effects and complications
Salah et al. (2018) [22]	Lung or pleural cancer	Thorax	Thoracic surgeries (GA)	Pregabalin (30): 300 mg PO	Control (30): placebo PO	Single	Morphine PCA	(1) Resting pain severity scores (2) Cumulative morphine equivalent consumption (3) Postoperative adverse effects and complications
Zhang et al. (2012) [35]	Breast cancer	Thorax	MRM (GA)	Pregabalin (37): 150 mg PO	Control (38): placebo PO	Single	Morphine IM	(1) Resting pain severity scores (2) Cumulative morphine equivalent consumption (3) Postoperative adverse effects and complications
Zhang (2016) [36]	Breast cancer	Thorax	MRM (GA)	Pregabalin (45): 150 mg PO	Control (45): placebo PO	Single	Morphine IM	(1) Dynamic pain severity scores (2) Cumulative morphine equivalent consumption

Abbreviation: *N*: number; SD: standard deviation; mg: milligram; GA: general anesthesia; LA: local anesthesia; RFA: radiofrequency ablation; MRM: modified radical mastectomy; ALND: axillary lymph nodes dissection; BCCS: breast-conserving cancer surgery; PO: peroral administration; N/A: not applicable; PCA: patient-controlled analgesia; IV: intravenous injection; IM: intramuscular injection.

**Table 2:** Endpoint results.

Outcome	*N* of Studies	Pregabalin, Mean (SD) or *n*/*N*	Control, Mean (SD) or *n*/*N*	WMD or RR (95% CI or 97.5% CI)	*p* Value for Statistical Significance	*p* Value for Heterogeneity	I^2^ Test Forheterogeneity	Quality of Evidence (GRADE)
**Primary indicators** Pain scores at 24 h postoperatively (cm)								
resting pain	9	1.66 (1.23)	2.09 (1.15)	−0.45 (−0.68 to −0.21)	<0.001	0.005	63.32%	⊕⊕◯◯
dynamic pain	7	2.53 (1.71)	2.72 (1.58)	−0.31 (−0.83 to 0.22)	0.19	<0.001	93.62%	⊕⊕◯◯
**Secondary indicators**								
Resting pain scores postoperatively (cm)								
at 1 h	2	1.97 (1.96)	4.01 (1.45)	−1.56 (−3.63 to 0.52)	0.14	<0.001	97%	⊕⊕⊕◯
at 2 h	7	1.80 (1.39)	3.56 (4.53)	−1.53 (−2.30 to −0.77)	<0.001	<0.001	97%	⊕⊕⊕◯
at 4 h	7	2.22 (1.39)	2.91 (1.16)	−0.53 (−0.98 to −0.08)	0.02	<0.001	92%	⊕⊕◯◯
at 6 h	7	2.54 (1.43)	3.44 (1.62)	−0.87 (−1.58 to −0.16)	0.02	<0.001	95%	⊕⊕⊕◯
at 8 h	4	1.86 (1.17)	2.72 (1.03)	−0.64 (−0.96 to −0.32)	<0.001	0.08	57%	⊕⊕◯◯
at 12 h	8	2.76 (1.43)	3.16 (1.25)	−0.59 (−1.06 to −0.12)	0.01	<0.001	88%	⊕⊕◯◯
at 16 h	3	1.41 (1.08)	2.57 (1.27)	−1.07 (−1.88 to −0.25)	0.01	<0.001	91%	⊕⊕◯◯
at 20 h	2	1.47 (1.05)	2.10 (0.82)	−0.61 (−1.18 to −0.05)	0.03	0.03	78%	⊕⊕◯◯
at 48 h	2	0.73 (0.89)	0.87 (0.57)	−0.13 (−0.42 to 0.15)	0.36	0.22	33%	⊕⊕⊕◯
Dynamic pain scores postoperatively (cm)								
at 2 h	5	2.94 (1.76)	4.41 (1.90)	−1.16 (−2.22 to −0.11)	0.03	<0.001	98%	⊕⊕⊕◯
at 4 h	4	3.04 (1.87)	3.89 (1.83)	−0.53 (−0.97 to −0.10)	0.02	<0.001	85%	⊕⊕◯◯
at 6 h	5	3.37 (2.02)	4.31 (2.04)	−1.03 (−1.83 to −0.23)	0.01	<0.001	95%	⊕⊕⊕◯
at 8 h	2	2.54 (1.19)	3.19 (1.06)	−0.36 (−0.78 to 0.06)	0.10	0.12	59%	⊕⊕◯◯
at 12 h	5	3.68 (2.07)	4.82 (1.57)	−0.85 (−1.49 to −0.21)	0.01	<0.001	90%	⊕⊕◯◯
at 16 h	2	2.47 (1.16)	2.97 (0.92)	−0.26 (−0.54 to 0.02)	0.07	0.37	0%	⊕⊕⊕◯
at 48 h	2	1.63 (1.31)	1.98 (1.34)	−0.31 (−1.17 to 0.54)	0.47	0.13	57%	⊕⊕◯◯
Cumulative morphine equivalent consumption (mg)								
within 12 h	2	8.46 (3.48)	11.36 (4.64)	−1.77 (−6.77 to 3.24)	0.49	0.001	90%	⊕⊕⊕◯
within 24 h	10	14.14 (12.71)	23.69 (22.17)	−7.45 (−9.30 to −5.60)	<0.001	<0.001	96%	⊕◯◯◯
within 48 h	2	23.01 (20.73)	63.70 (48.14)	−29.93 (−81.99 to 22.13)	0.26	<0.001	97%	⊕◯◯◯
Time to first analgesic request (hours)								
Time to first analgesic request	4	5.02 (3.68)	1.97 (1.50)	2.28 (0.79 to 3.77)	0.003	<0.001	100%	⊕◯◯◯
Hemodynamic parameters								
Heart rate (beat/min)								
at 2 h	2	84.05 (11.98)	87.02 (14.80)	−2.81 (−7.71 to 2.08)	0.26	0.79	0%	⊕⊕⊕⊕
at 6 h	2	83.25 (11.08)	86.06 (15.79)	−2.53 (−7.59 to 2.53)	0.33	0.64	0%	⊕⊕⊕⊕
at 12 h	2	83.20 (10.34)	87.37 (15.00)	−3.83 (−8.59 to 0.92)	0.11	0.56	0%	⊕⊕⊕⊕
at 24 h	2	82.70 (11.24)	85.62 (14.45)	−2.73 (−7.44 to 1.98)	0.26	0.74	0%	⊕⊕⊕⊕
SBP (mm/Hg)								
at 2 h	2	105.62 (25.56)	116.33 (24.64)	−9.14 (−19.88 to 1.59)	0.10	0.15	51%	⊕⊕⊕◯
at 6 h	2	122.31 (12.56)	123.96 (12.74)	−1.79 (−6.31 to 2.72)	0.44	0.65	0%	⊕⊕⊕⊕
at 12 h	2	127.13 (15.10)	122.98 (12.46)	3.31 (−5.59 to 12.22)	0.47	0.09	66%	⊕⊕⊕◯
at 24 h	2	114.70 (15.68)	114.50 (14.23)	0.25 (−4.59 to 5.08)	0.92	0.52	0%	⊕⊕⊕⊕
DBP (mm/Hg)								
at 2 h	2	76.03 (10.58)	76.33 (11.22)	−0.13 (−4.06 to 3.81)	0.95	0.29	9%	⊕⊕⊕⊕
at 6 h	2	78.51 (10.85)	80.82 (9.93)	−2.26 (−5.45 to 0.93)	0.16	0.73	0%	⊕⊕⊕⊕
at 12 h	2	82.70 (12.09)	79.18 (10.32)	1.99 (−8.60 to 12.58)	0.71	0.004	88%	⊕⊕⊕◯
at 24 h	2	73.93 (10.34)	74.59 (9.78)	−1.55 (−6.59 to 3.50)	0.55	0.13	57%	⊕⊕⊕◯
**The safety indicators**								
Pregabalin-related side effects								
Dizziness	7	70/293	18/197	2.81 (1.75 to 4.53)	<0.001	0.47	0%	⊕⊕⊕◯
Visual disturbance	6	43/256	6/159	3.04 (1.37 to 6.73)	0.006	0.64	0%	⊕⊕⊕◯
Pruritus	4	0/127	4/93	0.14 (0.02 to 1.02)	0.05	0.49	0%	⊕⊕⊕◯
Headache	4	33/196	11/99	1.71 (0.88 to 3.31)	0.25	0.11	26%	⊕⊕⊕⊕
Sedation score at 12 h	3	1.35 (1.00)	1.18 (0.87)	0.35 (0.15 to 0.55)	<0.001	N/A	N/A	⊕⊕⊕⊕
Sedation score at 24 h	3	1.21 (1.07)	0.99 (0.92)	0.50 (0.15 to 0.86)	0.006	0.13	57%	⊕⊕⊕◯
Opioid-related side effects								
PONV	10	86/393	103/277	0.59 (0.39 to 0.87)	0.008	0.01	60%	⊕⊕◯◯

⊕⊕⊕⊕, high-quality evidence; ⊕⊕⊕◯, moderate-quality evidence; ⊕⊕◯◯, low-quality evidence, ⊕◯◯◯, very-low-quality evidence. Abbreviations: SD: standard deviation; WMD: weighted mean difference; RR: risk ratio; CI: confidence interval; GRADE: Grades of Recommendation, Assessment, Development, and Evaluation; cm: centimeter; mg: milligrams; N/A: not applicable; I^2^: I-square; SBP: systolic blood pressure; DBP: diastolic blood pressure; PONV: postoperative nausea and vomiting.

## Data Availability

The raw data and statistical code for conducting this meta-analysis is available upon request.

## Supplementary Materials

The following supporting information can be downloaded at: https://www.mdpi.com/article/10.3390/medicina59020280/s1, Methods S1: PubMed Search Strategy; Methods S2: Web of Science Search Strategy; Methods S3: EMBASE Search Strategy; Methods S4: Cochrane Library Search Strategy; Methods S5: Google Scholar Search Strategy; Table S1: Risk of Bias Assessment; Table S2: GRADE quality of evidence summary table; Table S3: Meta-regression analysis for primary outcomes; Table S4: (A) Subgroup analyses for primary and safety outcomes; (B) Predefined sensitivity analyses for primary outcomes; Figure S1: Risk of bias summary.

## References

[1] Horowitz M., Neeman E., Sharon E., Ben-Eliyahu S. (2015). Exploiting the critical perioperative period to improve long-term cancer outcomes. Nat. Rev. Clin. Oncol..

[2] Chang S.H., Mehta V., Langford R.M. (2009). Acute and chronic pain following breast surgery. Acute Pain.

[3] Wu C.L., Naqibuddin M., Rowlingson A.J., Lietman S.A., Jermyn R.M., Fleisher L.A. (2003). The effect of pain on health-related quality of life in the immediate postoperative period. Anesth. Analg..

[4] Silver J.K., Baima J., Mayer R.S. (2013). Impairment-driven cancer rehabilitation: An essential component of quality care and survivorship. CA Cancer J. Clin..

[5] Trakimas D.R., Perez-Heydrich C., Mandal R., Tan M., Gourin C.G., Fakhry C., Koch W.M., Russell J.O., Tufano R.P., Eisele D.W. (2022). Peri-Operative Pain and Opioid Use in Opioid-Naïve Patients Following Inpatient Head and Neck Surgery. Front. Psychiatry.

[6] Karlsdottir B.R., Zhou P.P., Wahba J., Mott S.L., Goffredo P., Hrabe J., Hassan I., Kapadia M.R., Gribovskaja-Rupp I. (2022). Male gender, smoking, younger age, and preoperative pain found to increase postoperative opioid requirements in 592 elective colorectal resections. Int. J. Colorectal Dis..

[7] Wiffen P.J., Wee B., Derry S., Bell R.F., Moore R.A. (2017). Opioids for cancer pain-an overview of Cochrane reviews. Cochrane Database Syst. Rev..

[8] Hill M.V., McMahon M.L., Stucke R.S., Barth R.J. (2017). Wide Variation and Excessive Dosage of Opioid Prescriptions for Common General Surgical Procedures. Ann. Surg..

[9] Brogi E., Forfori F. (2022). Anesthesia and cancer recurrence: An overview. J. Anesth. Analg. Crit. Care.

[10] Moorthy A., Eochagáin A.N., Buggy D.J. (2021). Can Acute Postoperative Pain Management After Tumour Resection Surgery Modulate Risk of Later Recurrence or Metastasis?. Front. Oncol..

[11] Diaz-Cambronero O., Mazzinari G., Cata J.P. (2018). Perioperative opioids and colorectal cancer recurrence: A systematic review of the literature. Pain Manag..

[12] Richebé P., Capdevila X., Rivat C. (2018). Persistent Postsurgical Pain: Pathophysiology and Preventative Pharmacologic Considerations. Anesthesiology.

[13] Kehlet H., Jensen T.S., Woolf C.J. (2006). Persistent postsurgical pain: Risk factors and prevention. Lancet.

[14] Chincholkar M. (2020). Gabapentinoids: Pharmacokinetics, pharmacodynamics and considerations for clinical practice. Br. J. Pain.

[15] Fonseca F., Lenahan W., Dart R.C., Papaseit E., Dargan P.I., Wood D.M., Guareschi M., Maremmani I., Auriacombe M., Farré M. (2021). Non-medical Use of Prescription Gabapentinoids (Gabapentin and Pregabalin) in Five European Countries. Front. Psychiatry.

[16] Kremer M., Yalcin I., Nexon L., Wurtz X., Ceredig R.A., Daniel D., Hawkes R.A., Salvat E., Barrot M. (2016). The antiallodynic action of pregabalin in neuropathic pain is independent from the opioid system. Mol. Pain..

[17] Chen Z., Chen J., Luo R., Jiang J., Xiang Z. (2022). The preemptive effects of oral pregabalin on perioperative pain management in lower limb orthopedic surgery: A systematic review and meta-analysis. J. Orthop. Surg. Res..

[18] Fabritius M.L., Strøm C., Koyuncu S., Jæger P., Petersen P.L., Geisler A., Wetterslev J., Dahl J.B., Mathiesen O. (2017). Benefit and harm of pregabalin in acute pain treatment: A systematic review with meta-analyses and trial sequential analyses. Br. J. Anaesth..

[19] Mishriky B.M., Waldron N.H., Habib A.S. (2015). Impact of pregabalin on acute and persistent postoperative pain: A systematic review and meta-analysis. Br. J. Anaesth..

[20] Liberati A., Altman D.G., Tetzlaff J., Mulrow C., Gøtzsche P.C., Ioannidis J.P., Clarke M., Devereaux P.J., Kleijnen J., Moher D. (2009). The PRISMA statement for reporting systematic reviews and meta-analyses of studies that evaluate healthcare interventions: Explanation and elaboration. BMJ.

[21] Cochrane Handbook for Systematic Reviews of Interventions Version 6.3. Cochrane. www.training.cochrane.org/handboo.

[22] Salah Abdelgalil A., Shoukry A.A., Kamel M.A., Heikal A.M.Y., Ahmed N.A. (2019). Analgesic Potentials of Preoperative Oral Pregabalin, Intravenous Magnesium Sulfate, and their Combination in Acute Postthoracotomy Pain. Clin. J. Pain..

[23] Sterne J.A.C., Savović J., Page M.J., Elbers R.G., Blencowe N.S., Boutron I., Cates C.J., Cheng H.Y., Corbett M.S., Eldridge S.M. (2019). RoB 2: A revised tool for assessing risk of bias in randomised trials. Bmj.

[24] Brozek J.L., Akl E.A., Alonso-Coello P., Lang D., Jaeschke R., Williams J.W., Phillips B., Lelgemann M., Lethaby A., Bousquet J. (2009). Grading quality of evidence and strength of recommendations in clinical practice guidelines. Part 1 of 3. An overview of the GRADE approach and grading quality of evidence about interventions. Allergy.

[25] Balshem H., Helfand M., Schünemann H.J., Oxman A.D., Kunz R., Brozek J., Vist G.E., Falck-Ytter Y., Meerpohl J., Norris S. (2011). GRADE guidelines: 3. Rating the quality of evidence. J. Clin. Epidemiol..

[26] Zhang Y., Coello P.A., Guyatt G.H., Yepes-Nuñez J.J., Akl E.A., Hazlewood G., Pardo-Hernandez H., Etxeandia-Ikobaltzeta I., Qaseem A., Williams J.W. (2019). GRADE guidelines: 20. Assessing the certainty of evidence in the importance of outcomes or values and preferences-inconsistency, imprecision, and other domains. J. Clin. Epidemiol..

[27] Earsakul A. (2017). Analgesic efficacy of preoperative administration of low-dose pregabalin in patients undergoing breast cancer surgery. Thai J. Anesthesiol..

[28] Ghoneim A.A., Hegazy M.M. (2013). The analgesic effect of preoperative pregabalin in radical cystectomy for cancer bladder patients. Chin.-Ger. J. Clin. Oncol..

[29] Hetta D.F., Mohamed M.A., Mohammad M.F. (2016). Analgesic efficacy of pregabalin in acute postmastectomy pain: Placebo controlled dose ranging study. J. Clin. Anesth..

[30] Lamsal R., Mahajan C., Chauhan V., Gupta N., Mishra N., Rath G.P. (2019). Effect of Pregabalin on Postcraniotomy Pain in Patients Undergoing Supratentorial Tumor Surgery: A Randomized, Double-Blind, Placebo-Controlled Trial. J. Neurosci. Rural. Pract..

[31] Mahran E., Hassan M.E. (2015). Comparison of pregabalin versus ketamine in postoperative pain management in breast cancer surgery. Saudi J. Anaesth..

[32] Mansor S.H., Choy C.Y. (2015). Effect of preoperative oral pregabalin on postoperative pain after mastectomy. Middle East. J. Anaesthesiol..

[33] Mohamed M.A., Othman A.H., Abd El-Rahman A.M. (2016). Analgesic efficacy and safety of peri-operative pregabalin following radical cystectomy: A dose grading study. Egypt. J. Anaesth..

[34] Patel P.M., Panchal R.D., Patel B.M. (2016). A randomized placebo controlled trial on evaluation of the efficacy of two different doses of pregabalin as a pre-emptive analgesia in gynaecological surgeries. Indian J. Clin. Anaesth..

[35] Zhang Q., Wang J.F., Liu E.Y., Wang Y.L. (2012). The effect Pregabalin on post operative pain control after modified mastectomy. Chin. J. Curr. Adv. Gen. Surg..

[36] Zhang A.C. (2016). Curative Effects of Preoperative Oral Pregabalin on Plasma Substance P and Beta Endorphinin Breast Cancer. Pract. J. Cancer.

[37] Rivasi G., Menale S., Turrin G., Coscarelli A., Giordano A., Ungar A. (2022). The Effects of Pain and Analgesic Medications on Blood Pressure. Curr. Hypertens. Rep..

[38] Lee C.R., McTavish D., Sorkin E.M. (1993). Tramadol. A preliminary review of its pharmacodynamic and pharmacokinetic properties, and therapeutic potential in acute and chronic pain states. Drugs.

[39] Poklis A. (1995). Fentanyl: A review for clinical and analytical toxicologists. J. Toxicol. Clin. Toxicol..

[40] Luo D., Wan X., Liu J., Tong T. (2018). Optimally estimating the sample mean from the sample size, median, mid-range, and/or mid-quartile range. Stat. Methods Med. Res..

[41] DerSimonian R., Laird N. (1986). Meta-analysis in clinical trials. Control Clin. Trials.

[42] Holm S. (1979). A simple sequentially rejective multiple test procedure. Scand. J. Stat..

[43] Ye X., Ren Y.F., Hu Y.C., Tan S.Y., Jiang H., Zhang L.F., Shi W., Wang Y.T. (2022). Dexamethasone Does Not Provide Additional Clinical Analgesia Effect to Local Wound Infiltration: A Comprehensive Systematic Review and Meta-analysis. Adv. Wound. Care.

[44] Hussain N., Brull R., Noble J., Weaver T., Essandoh M., McCartney C.J., Abdallah F.W. (2021). Statistically significant but clinically unimportant: A systematic review and meta-analysis of the analgesic benefits of erector spinae plane block following breast cancer surgery. Reg. Anesth. Pain Med..

[45] Doleman B., Leonardi-Bee J., Heinink T.P., Boyd-Carson H., Carrick L., Mandalia R., Lund J.N., Williams J.P. (2021). Pre-emptive and preventive NSAIDs for postoperative pain in adults undergoing all types of surgery. Cochrane Database Syst. Rev..

[46] Egger M., Davey Smith G., Schneider M., Minder C. (1997). Bias in meta-analysis detected by a simple, graphical test. BMJ.

[47] SK P.G.R., Bhagyashree A. (2016). Efficacy of Preemptive oral pregabalin for prolonging post-operative analgesia in modified radical mastectomies. Indian J. Clin. Anaesth..

[48] Pushkarna G., Badhan C., Gupta R., Chawla S., Abbi P. (2022). Evaluation of the postoperative morphine-sparing effect of oral premedicants used as pre-emptive analgesics in breast-conserving cancer surgeries: A randomised placebo-controlled trial. Indian J. Anaesth..

[49] Chen H.H., Li Y.D., Cheng P.W., Fang Y.C., Lai C.C., Tseng C.J., Pan J.Y., Yeh T.C. (2019). Gabapentin Reduces Blood Pressure and Heart Rate through the Nucleus Tractus Solitarii. Acta Cardiol. Sin..

[50] Chen W., Huang H., Yang C., Hu X., Bao F., Jiang H. (2019). Preoperative Low-dose and High-dose Pregabalin and Cardiovascular Response to Endotracheal Intubation: A Prospective, Randomized, Single-blind, Controlled Study in China. Clin. Ther..

[51] Cozzolino D., Sasso F.C., Cataldo D., Gruosso D., Giammarco A., Cavalli A., Di Maggio C., Renzo G., Salvatore T., Giugliano D. (2005). Acute pressor and hormonal effects of beta-endorphin at high doses in healthy and hypertensive subjects: Role of opioid receptor agonism. J. Clin. Endocrinol. Metab..

[52] Bugan I., Karagoz Z., Altun S., Djamgoz M.B. (2016). Gabapentin, an Analgesic Used Against Cancer-Associated Neuropathic Pain: Effects on Prostate Cancer Progression in an In Vivo Rat Model. Basic Clin. Pharmacol Toxicol..

[53] Pellegrino M., Ricci E., Ceraldi R., Nigro A., Bonofiglio D., Lanzino M., Morelli C. (2022). From HDAC to Voltage-Gated Ion Channels: What&rsquo;s Next? The Long Road of Antiepileptic Drugs Repositioning in Cancer. Cancers.

[54] Grasso A., Orsaria P., Costa F., D’Avino V., Caredda E., Hazboun A., Carino R., Pascarella G., Altomare M., Buonomo O.C. (2020). Ultrasound-guided Interfascial Plane Blocks for Non-anesthesiologists in Breast Cancer Surgery: Functional Outcomes and Benefits. Anticancer Res..

[55] Costa F., Strumia A., Remore L.M., Pascarella G., Del Buono R., Tedesco M., Sepolvere G., Scimia P., Fusco P. (2020). Breast surgery analgesia: Another perspective for PROSPECT guidelines. Anaesthesia.

[56] Fabio C., Giuseppe P., Chiara P., Antongiulio V., Enrico D.S., Filippo R., Federica B., Eugenio A.F. (2019). Sufentanil sublingual tablet system (Zalviso(®)) as an effective analgesic option after thoracic surgery: An observational study. Saudi J. Anaesth..

[57] Zhang J., Ho K.Y., Wang Y. (2011). Efficacy of pregabalin in acute postoperative pain: A meta-analysis. Br. J. Anaesth..

[58] Engelman E., Cateloy F. (2011). Efficacy and safety of perioperative pregabalin for post-operative pain: A meta-analysis of randomized-controlled trials. Acta Anaesthesiol. Scand..

[59] Zhang Y., Wang Y., Zhang X. (2017). Effect of pre-emptive pregabalin on pain management in patients undergoing laparoscopic cholecystectomy: A systematic review and meta-analysis. Int. J. Surg..

[60] Dong J., Li W., Wang Y. (2016). The effect of pregabalin on acute postoperative pain in patients undergoing total knee arthroplasty: A meta-analysis. Int. J. Surg..

[61] Chang C.C., Yen W.T., Lin Y.T., Wang L.K., Hung K.C., Wu Z.F., Chen J.Y. (2020). Perioperative Pregabalin for Preventive Analgesia in Breast Cancer Surgery: A Meta-analysis of Randomized Controlled Trials. Clin. J. Pain.

[62] Rai A.S., Khan J.S., Dhaliwal J., Busse J.W., Choi S., Devereaux P.J., Clarke H. (2017). Preoperative pregabalin or gabapentin for acute and chronic postoperative pain among patients undergoing breast cancer surgery: A systematic review and meta-analysis of randomized controlled trials. J. Plast. Reconstr. Aesthet. Surg..

[63] Verret M., Lauzier F., Zarychanski R., Perron C., Savard X., Pinard A.M., Leblanc G., Cossi M.J., Neveu X., Turgeon A.F. (2020). Perioperative Use of Gabapentinoids for the Management of Postoperative Acute Pain: A Systematic Review and Meta-analysis. Anesthesiology.

[64] Carr D.B., Goudas L.C. (1999). Acute pain. Lancet.

[65] Durkin B., Page C., Glass P. (2010). Pregabalin for the treatment of postsurgical pain. Expert. Opin. Pharmacother..

[66] Ben-Menachem E. (2004). Pregabalin pharmacology and its relevance to clinical practice. Epilepsia.

