# Supplementary material for: A Meta-Analysis Showing the Quantitative Evidence Base of Preemptive Pregabalin for Postoperative Pain from Cancer-Related Surgery
Source: Medicina (Kaunas). 2023 Jan 31;59(2):280. doi: 10.3390/medicina59020280 (PMC9965191; doi:10.3390/medicina59020280)

Supplementary Online Content

Table of Contents	Page
**Methods S1:** PubMed Search Strategy Search strategy	**2-5**
**Methods S2:** Web of Science Search Strategy Search strategy	**6**
**Methods S3:** EMBASE Search Strategy Search strategy	**6**
**Methods S4:** Cochrane Library Search Strategy Search strategy	**7**
**Methods S5:** Google Scholar Search Strategy Search strategy	**8**
**Table S1:** Risk of Bias Assessment	**9-16**
**Table S2:** GRADE quality of evidence summary table	**17-29**
**Table S3:** Meta-regression analysis for primary outcomes	**30**
**Table S4A:** Subgroup analyses for primary and safety outcomes	**31-33**
**Table S4B:** Predefined sensitivity analyses for primary outcomes	**34**
**Figure S1:** Risk of bias summary	**35**

Methods S1: PubMed Search Strategy Search strategy

PubMed (48)

**Subject words:**	**Free words**
Pregabalin	(S)-3-(aminomethyl)-5-methylhexanoic acid<br>3-isobutyl GABA<br>3 isobutyl GABA<br>GABA, 3-isobutyl<br>3-(aminomethyl)-5-methylhexanoic acid<br>(R-)-3-isobutyl GABA<br>(S+)-3-isobutyl GABA<br>Lyrica<br>CI 1008<br>1008, CI<br>CI-1008<br>CI1008<br>gamma-Aminobutyric Acid<br>GABA
Neoplasms	Tumor<br>Neoplasm<br>Tumors<br>Neoplasia<br>Neoplasias<br>Cancer<br>Cancers<br>Malignant Neoplasm<br>Malignancy<br>Malignancies<br>Malignant Neoplasms<br>Neoplasm, Malignant<br>Neoplasms, Malignant<br>Benign Neoplasms<br>Benign Neoplasm<br>Neoplasms, Benign<br>Neoplasm, Benign
General Surgery<br>Surgical Procedures, Operative	operative therapy<br>invasive procedures<br>operative procedures

	operations<br>perioperative procedures<br>intraoperative procedures<br>peroperative procedures<br>preoperative procedures<br>Surgery<br>Surgery, General

1. ((((((((((((((Pregabalin[MeSH Terms]) OR (Pregabalin[Title/Abstract])) OR ((S)-3-(aminomethyl)-5-methylhexanoic acid[Title/Abstract])) OR (3-isobutyl GABA[Title/Abstract])) OR (3 isobutyl GABA[Title/Abstract])) OR (GABA, 3-isobutyl[Title/Abstract])) OR (3-(aminomethyl)-5-methylhexanoic acid[Title/Abstract])) OR ((R-)-3-isobutyl GABA[Title/Abstract])) OR ((S+)-3-isobutyl GABA[Title/Abstract])) OR (Lyrica[Title/Abstract])) OR (CI 1008[Title/Abstract])) OR (1008, CI[Title/Abstract])) OR (CI-1008[Title/Abstract])) OR (CI1008[Title/Abstract])) OR (gamma-Aminobutyric Acid[Title/Abstract])) OR (GABA[Title/Abstract]) **[70,776]**

2. ((((((((((((((((((((Neoplasms[MeSH Terms])) OR (Neoplasms[Title/Abstract])) OR (Tumor[Title/Abstract])) OR (Neoplasm[Title/Abstract])) OR (Tumors[Title/Abstract])) OR (Neoplasia[Title/Abstract])) OR (Neoplasias[Title/Abstract])) OR (Cancer[Title/Abstract])) OR (Cancers[Title/Abstract])) OR (Malignant Neoplasm[Title/Abstract])) OR (Malignancy[Title/Abstract])) OR (Malignancies[Title/Abstract])) OR (Malignant Neoplasms[Title/Abstract])) OR (Neoplasm, Malignant[Title/Abstract])) OR (Neoplasms, Malignant[Title/Abstract])) OR (Benign Neoplasms[Title/Abstract])) OR (Benign Neoplasm[Title/Abstract])) OR (Neoplasms, Benign[Title/Abstract])) OR (Neoplasm, Benign[Title/Abstract])) OR (cancer)) OR (tumor) **[5,202,337]**

3. ((Surgical Procedures, Operative[MeSH Terms]) OR ((((((((((((General Surgery[MeSH Terms]) OR (Surgery[MeSH Subheading])) OR (General Surgery[Title/Abstract])) OR (Surgery[Title/Abstract])) OR (operative therapy[Title/Abstract])) OR (invasive procedures[Title/Abstract])) OR (operative procedures[Title/Abstract])) OR (operations[Title/Abstract])) OR (perioperative procedures[Title/Abstract])) OR (intraoperative procedures[Title/Abstract])) OR (peroperative procedures[Title/Abstract])) OR (preoperative procedures[Title/Abstract])) OR (Surgery, General[Title/Abstract])) OR (Preoperative[Title/Abstract]) **[4,658,132]**

4. random [**1,365,784**]

5. (((((((((((((((((Pregabalin[MeSH Terms]) OR (Pregabalin[Title/Abstract])) OR ((S)-3-(aminomethyl)-5-methylhexanoic acid[Title/Abstract])) OR (3-isobutyl

GABA[Title/Abstract])) OR (3 isobutyl GABA[Title/Abstract])) OR (GABA, 3-isobutyl[Title/Abstract])) OR (3-(aminomethyl)-5-methylhexanoic acid[Title/Abstract])) OR ((R-)-3-isobutyl GABA[Title/Abstract])) OR ((S+)-3-isobutyl GABA[Title/Abstract])) OR (Lyrica[Title/Abstract])) OR (CI 1008[Title/Abstract])) OR (1008, CI[Title/Abstract])) OR (CI-1008[Title/Abstract])) OR (CI1008[Title/Abstract])) OR (gamma-Aminobutyric Acid[Title/Abstract])) OR (GABA[Title/Abstract])) AND (((((((((((((((((((((((Neoplasms[MeSH Terms])) OR (Neoplasms[Title/Abstract])) OR (Tumor[Title/Abstract])) OR (Neoplasm[Title/Abstract])) OR (Tumors[Title/Abstract])) OR (Neoplasia[Title/Abstract])) OR (Neoplasias[Title/Abstract])) OR (Cancer[Title/Abstract])) OR (Cancers[Title/Abstract])) OR (Malignant Neoplasm[Title/Abstract])) OR (Malignancy[Title/Abstract])) OR (Malignancies[Title/Abstract])) OR (Malignant Neoplasms[Title/Abstract])) OR (Neoplasm, Malignant[Title/Abstract])) OR (Neoplasms, Malignant[Title/Abstract])) OR (Benign Neoplasms[Title/Abstract])) OR (Benign Neoplasm[Title/Abstract])) OR (Neoplasms, Benign[Title/Abstract])) OR (Neoplasm, Benign[Title/Abstract])) OR (cancer)) OR (tumor))) AND (((Surgical Procedures, Operative[MeSH Terms]) OR ((((((((((((General Surgery[MeSH Terms]) OR (Surgery[MeSH Subheading])) OR (General Surgery[Title/Abstract])) OR (Surgery[Title/Abstract])) OR (operative therapy[Title/Abstract])) OR (invasive procedures[Title/Abstract])) OR (operative procedures[Title/Abstract])) OR (operations[Title/Abstract])) OR (perioperative procedures[Title/Abstract])) OR (intraoperative procedures[Title/Abstract])) OR (peroperative procedures[Title/Abstract])) OR (preoperative procedures[Title/Abstract])) OR (Surgery, General[Title/Abstract])) OR (Preoperative[Title/Abstract]))) AND (random) [**48**]

Search	Actions	Details	Query	Results	Time
#5	•••	>	Search: **((((((((((((((((Pregabalin[MeSH Terms]) OR (Pregabalin[Title/Abstract])) OR ((S)-3-(aminomethyl)-5-methylhexanoic acid[Title/Abstract])) OR (3-isobutyl GABA[Title/Abstract])) OR (3 isobutyl GABA[Title/Abstract])) OR (GABA, 3-isobutyl[Title/Abstract])) OR (3-(aminomethyl)-5-methylhexanoic acid[Title/Abstract])) OR ((R-)-3-isobutyl GABA[Title/Abstract])) OR ((S+)-3-isobutyl GABA[Title/Abstract])) OR (Lyrica[Title/Abstract])) OR (CI 1008[Title/Abstract])) OR (1008, CI[Title/Abstract])) OR (CI-1008[Title/Abstract])) OR (CI1008[Title/Abstract])) OR (gamma-Aminobutyric Acid[Title/Abstract])) OR (GABA[Title/Abstract])) AND ((((((((((((((((((((Neoplasms[MeSH Terms])) OR (Neoplasms[Title/Abstract])) OR (Tumor[Title/Abstract])) OR (Neoplasm[Title/Abstract])) OR (Tumors[Title/Abstract])) OR (Neoplasia[Title/Abstract])) OR (Neoplasias[Title/Abstract])) OR (Cancer[Title/Abstract])) OR (Cancers[Title/Abstract])) OR (Malignant Neoplasm[Title/Abstract])) OR (Malignancy[Title/Abstract])) OR (Malignancies[Title/Abstract])) OR (Malignant Neoplasms[Title/Abstract])) OR (Neoplasm, Malignant[Title/Abstract])) OR (Neoplasms, Malignant[Title/Abstract])) OR (Benign Neoplasms[Title/Abstract])) OR (Benign Neoplasm[Title/Abstract])) OR (Neoplasms, Benign[Title/Abstract])) OR (Neoplasm, Benign[Title/Abstract])) OR (cancer)) OR (tumor))) AND (((Surgical Procedures, Operative[MeSH Terms]) OR (((((((((((General Surgery[MeSH Terms]) OR (Surgery[MeSH Subheading])) OR (General Surgery[Title/Abstract])) OR (Surgery[Title/Abstract])) OR (operative therapy[Title/Abstract])) OR (invasive procedures[Title/Abstract])) OR (operative procedures[Title/Abstract])) OR (operations[Title/Abstract])) OR (perioperative procedures[Title/Abstract])) OR (intraoperative procedures[Title/Abstract])) OR (peroperative procedures[Title/Abstract])) OR (preoperative procedures[Title/Abstract])) OR (Surgery, General[Title/Abstract])) OR (Preoperative[Title/Abstract]))) AND (random)** Sort by: **Most Recent**	48	21:46:35
#4	•••	>	Search: **random** Sort by: **Most Recent**	1,365,784	20:50:31
#3	•••	>	Search: **((Surgical Procedures, Operative[MeSH Terms]) OR (((((((((((General Surgery[MeSH Terms]) OR (Surgery[MeSH Subheading])) OR (General Surgery[Title/Abstract])) OR (Surgery[Title/Abstract])) OR (operative therapy[Title/Abstract])) OR (invasive procedures[Title/Abstract])) OR (operative procedures[Title/Abstract])) OR (operations[Title/Abstract])) OR (perioperative procedures[Title/Abstract])) OR (intraoperative procedures[Title/Abstract])) OR (peroperative procedures[Title/Abstract])) OR (preoperative procedures[Title/Abstract])) OR (Surgery, General[Title/Abstract])) OR (Preoperative[Title/Abstract])** Sort by: **Most Recent**	4,658,132	20:50:15
#2	•••	>	Search: **((((((((((((((((((((Neoplasms[MeSH Terms])) OR (Neoplasms[Title/Abstract])) OR (Tumor[Title/Abstract])) OR (Neoplasm[Title/Abstract])) OR (Tumors[Title/Abstract])) OR (Neoplasia[Title/Abstract])) OR (Neoplasias[Title/Abstract])) OR (Cancer[Title/Abstract])) OR (Cancers[Title/Abstract])) OR (Malignant Neoplasm[Title/Abstract])) OR (Malignancy[Title/Abstract])) OR (Malignancies[Title/Abstract])) OR (Malignant Neoplasms[Title/Abstract])) OR (Neoplasm, Malignant[Title/Abstract])) OR (Neoplasms, Malignant[Title/Abstract])) OR (Benign Neoplasms[Title/Abstract])) OR (Benign Neoplasm[Title/Abstract])) OR (Neoplasms, Benign[Title/Abstract])) OR (Neoplasm, Benign[Title/Abstract])) OR (cancer)) OR (tumor)** Sort by: **Most Recent**	5,202,337	20:50:00
#1	•••	>	Search: **((((((((((((((Pregabalin[MeSH Terms]) OR (Pregabalin[Title/Abstract])) OR ((S)-3-(aminomethyl)-5-methylhexanoic acid[Title/Abstract])) OR (3-isobutyl GABA[Title/Abstract])) OR (3 isobutyl GABA[Title/Abstract])) OR (GABA, 3-isobutyl[Title/Abstract])) OR (3-(aminomethyl)-5-methylhexanoic acid[Title/Abstract])) OR ((R-)-3-isobutyl GABA[Title/Abstract])) OR ((S+)-3-isobutyl GABA[Title/Abstract])) OR (Lyrica[Title/Abstract])) OR (CI 1008[Title/Abstract])) OR (1008, CI[Title/Abstract])) OR (CI-1008[Title/Abstract])) OR (CI1008[Title/Abstract])) OR (gamma-Aminobutyric Acid[Title/Abstract])) OR (GABA[Title/Abstract])** Sort by: **Most Recent**	70,776	20:48:59

Web of Science (251)

#1: (((((((((((((TS=(Pregabalin)) OR TS=((S)-3-(aminomethyl)-5-methylhexanoic acid)) OR TS=(3-isobutyl GABA)) OR TS=(3 isobutyl GABA)) OR TS=(GABA, 3-isobutyl)) OR TS=(3-(aminomethyl)-5-methylhexanoic acid)) OR TS=((R-)-3-isobutyl GABA)) OR TS=((S+)-3-isobutyl GABA)) OR TS=(Lyrica)) OR TS=(CI 1008)) OR TS=(1008, CI)) OR TS=(CI-1008)) OR TS=(CI1008))) [**23,678**]

#2: ((((((((((((((((TS=(Neoplasms)) OR TS=(Tumor)) OR TS=(Neoplasm)) OR TS=(Tumors)) OR TS=(Neoplasia)) OR TS=(Neoplasias)) OR TS=(Cancer)) OR TS=(Cancers)) OR TS=(Malignant Neoplasm)) OR TS=(Malignancy)) OR TS=(Malignancies)) OR TS=(Malignant Neoplasms)) OR TS=(Neoplasm, Malignant)) OR TS=(Neoplasms, Malignant)) OR TS=(Benign Neoplasms)) OR TS=(Benign Neoplasm)) OR TS=(Neoplasms, Benign)) OR TS=(Neoplasm, Benign) [**8,645,674**]

#3: (((((((((TS=(General Surgery)) OR TS=(operative therapy)) OR TS=(invasive procedures)) OR TS=(operative procedures)) OR TS=(operations)) OR TS=(perioperative procedures)) OR TS=(intraoperative procedures)) OR TS=(peroperative procedures)) OR TS=(preoperative procedures)) OR TS=(Surgery)) OR TS=(Surgery, General) [**13,056,182**]

#4: #3 AND #2 AND #1 [190]

4	#3 AND #2 AND #1	190	Add to query
3	(((((((((TS=(General Surgery)) OR TS=(operative therapy)) OR TS=(invasive procedures)) OR TS=(operative procedures)) OR TS=(operations)) OR TS=(perioperative procedures)) OR TS=(intraoperative procedures)) OR TS=(peroperative procedures)) OR TS=(preoperative procedures)) OR TS=(Surgery)) OR TS=(Surgery, General)	13,056,182	Add to query
2	((((((((((((((((TS=(Neoplasms)) OR TS=(Tumor)) OR TS=(Neoplasm)) OR TS=(Tumors)) OR TS=(Neoplasia)) OR TS=(Neoplasias)) OR TS=(Cancer)) OR TS=(Cancers)) OR TS=(Malignant Neoplasm)) OR TS=(Malignancy)) OR TS=(Malignancies)) OR TS=(Malignant Neoplasms)) OR TS=(Neoplasm, Malignant)) OR TS=(Neoplasms, Malignant)) OR TS=(Benign Neoplasms)) OR TS=(Benign	8,645,674	Add to query
1	(((((((((((((TS=(Pregabalin)) OR TS=((S)-3-(aminomethyl)-5-methylhexanoic acid)) OR TS=(3-isobutyl GABA)) OR TS=(3 isobutyl GABA)) OR TS=(GABA, 3-isobutyl)) OR TS=(3-(aminomethyl)-5-methylhexanoic acid)) OR TS=((R-)-3-isobutyl GABA)) OR TS=((S+)-3-isobutyl GABA)) OR TS=(lyrics)) OR TS=(CI 1008)) OR TS=(1008, CI)) OR TS=(CI-1008)) OR TS=(cb1008)))	23,678	Add to query

Embase (1231)

History Save | Delete | Print view | Export | Email Combine > using And Or ^ Collapse

#4	#1 AND #2 AND #3	1,231
#3	'general surgery'/exp OR 'general surgery':ab,ti OR 'operative therapy':ab,ti OR 'invasive procedures':ab,ti OR 'operative procedures':ab,ti OR 'operations':ab,ti OR 'perioperative procedures':ab,ti OR 'intraoperative procedures':ab,ti OR 'peroperative procedures':ab,ti OR 'preoperative procedures':ab,ti OR 'surgery':ab,ti OR 'surgery, general':ab,ti OR 'surgical procedures, operative':ab,ti OR 'surgery'/exp	6,103,707
#2	'neoplasm'/exp OR 'tumor':ab,ti OR 'neoplasm':ab,ti OR 'tumors':ab,ti OR 'neoplasia':ab,ti OR 'neoplasias':ab,ti OR 'cancer':ab,ti OR 'cancers':ab,ti OR 'malignant neoplasm':ab,ti OR 'malignancy':ab,ti OR 'malignancies':ab,ti OR 'malignant neoplasms':ab,ti OR 'neoplasm, malignant':ab,ti OR 'neoplasms, malignant':ab,ti OR 'benign neoplasms':ab,ti OR 'benign neoplasm':ab,ti OR 'neoplasms, benign':ab,ti OR 'neoplasm, benign':ab,ti	6,422,779
#1	'pregabalin'/exp OR 'pregabalin':ab,ti OR '(s)-3-(aminomethyl)-5-methylhexanoic acid':ab,ti OR '3-isobutyl gaba':ab,ti OR '3 isobutyl gaba':ab,ti OR 'gaba, 3-isobutyl':ab,ti OR '3-(aminomethyl)-5-methylhexanoic acid':ab,ti OR '(r-)-3-isobutyl gaba':ab,ti OR '(s+)-3-isobutyl gaba':ab,ti OR 'lyrica':ab,ti OR 'ci 1008':ab,ti OR '1008, ci':ab,ti OR 'ci-1008':ab,ti OR 'ci1008':ab,ti OR 'gamma-aminobutyric acid':ab,ti OR 'gaba':ab,ti	93,405

Cochrane Library (46)

#1	MeSH descriptor: [Pregabalin] explode all trees	MeSH	888
#2	(Pregabalin):ti,ab,kw OR (3 isobutyl GABA):ti,ab,kw OR (Lyrica):ti,ab,kw OR (CI 1008):ti,ab,kw OR (1008, CI):ti,ab,kw	S Limits	2456
#3	(CI-1008):ti,ab,kw OR (CI1008):ti,ab,kw OR (gamma-Aminobutyric Acid):ti,ab,kw OR (GABA):ti,ab,kw	S Limits	3137
#4	#1 or #2 or #3	Limits	5237
#5	MeSH descriptor: [Neoplasms] explode all trees	MeSH	87970
#6	(Neoplasms):ti,ab,kw OR (Tumor):ti,ab,kw OR (Neoplasm):ti,ab,kw OR (Tumors):ti,ab,kw OR (Neoplasia):ti,ab,kw	S Limits	141384
#7	(Neoplasias):ti,ab,kw OR (Cancer):ti,ab,kw OR (Cancers):ti,ab,kw OR (Malignant Neoplasm):ti,ab,kw OR (Malignancy):ti,ab,kw	S Limits	184068
#8	(Malignancies):ti,ab,kw OR (Malignant Neoplasms):ti,ab,kw OR (Neoplasm, Malignant):ti,ab,kw OR (Neoplasms, Malignant):ti,ab,kw OR (Benign [illegible]	S Limits	16863
#9	(Benign Neoplasm):ti,ab,kw OR (Neoplasms, Benign):ti,ab,kw OR (Neoplasm, Benign):ti,ab,kw	S Limits	3276
#10	#5 or #6 or #7 or #8 or #9	Limits	239165
#11	MeSH descriptor: [General Surgery] explode all trees	MeSH	366
#12	(General Surgery):ti,ab,kw OR (operative therapy):ti,ab,kw OR (invasive procedures):ti,ab,kw OR (operative procedures):ti,ab,kw OR (operations):ti,ab,kw	S Limits	55290
#13	(perioperative procedures):ti,ab,kw OR (intraoperative procedures):ti,ab,kw OR (peroperative procedures):ti,ab,kw OR (preoperative procedures):ti,ab,kw OR (Surgery):ti,ab,kw	S Limits	236390
#14	(Surgery, General):ti,ab,kw	S Limits	26510
#15	MeSH descriptor: [Surgical Procedures, Operative] explode all trees	MeSH	128153
#16	#11 or #12 or #13 or #14 or #15	Limits	295723
#17	#4 and #10 and # 16	Limits	46

Google Scholar (100)

intitle: (Pregabalin) AND (Neoplasms OR Tumor OR Cancer) AND (General Surgery OR operative therapy OR Surgery OR Surgical Procedures, Operative) AND randomized

Ten pages of results (100 citations) included.

CNKI (36)

Wan-Fang database (23)

Table S1: Risk of Bias Assessment
1. Earsakul 2017
RoB 2 tool

Bias	Author's judgement	Support for judgement
Bias arising from the randomization process	Low risk	Using a computer-generated random number table. Study medications were prepared in identical capsules, packed and sealed in opaque containers with labeled randomization number.
Bias due to deviations from intended interventions	Low risk	Double-blinded. Study medications were prepared in identical capsules, packed and sealed in opaque containers. The amount of intravenous agent and volatile agent were titrated by the attending anesthesiologist who was blinded to the randomization.
Bias due to missing outcome data	Low risk	Analyses included almost all subjects. The exclusion of patients did not affect the study result.
Bias in measurement of the outcome	Low risk	The method of measuring was appropriate. Per protocol and intention to treat were used to analyze the missing data.
Bias in selection of the reported result	Low risk	Pre-specified outcomes reported.
Overall bias	Low risk	The overall risk of bias was classified as low risk.

2. Ghoneim 2013
RoB 2 tool

Bias	Author's judgement	Support for judgement
Bias arising from the randomization process	Some concerns	Using a computer generated random numbers table.
Bias due to deviations from intended interventions	High risk	Patients were not blinded. All measurements were recorded by the same resident in charge who was blinded to the study drugs administered.

Bias due to missing outcome data	Low risk	Analyses included almost all subjects. The exclusion of patients did not affect the study result.
Bias in measurement of the outcome	Low risk	The method of measuring was appropriate.
Bias in selection of the reported result	Low risk	Pre-specified outcomes reported.
Overall bias	High risk	The overall risk of bias was classified as high risk.

3. Hetta 2016
RoB 2 tool

Bias	Author's judgement	Support for judgement
Bias arising from the randomization process	Low risk	Using a computer-generated random number list.
Bias due to deviations from intended interventions	Low risk	The study drugs were packed in opaque plastic containers labeled with the randomization numbers. They masked the study medication by packing placebo and pregabalin into 2 identical capsules in color and appearance to make the drugs unrecognizable.
Bias due to missing outcome data	Low risk	Analyses included almost all subjects. The exclusion of patients did not affect the study result.
Bias in measurement of the outcome	Low risk	The method of measuring was appropriate. They masked the study medication by packing placebo and pregabalin into 2 identical capsules in color and appearance to make the drugs unrecognizable.
Bias in selection of the reported result	Low risk	Pre-specified outcomes reported.
Overall bias	Low risk	The overall risk of bias was classified as low risk.

4. Lamsal 2019

RoB 2 tool

Bias	Author's judgement	Support for judgement
Bias arising from the randomization process	Low risk	Using a computer-generated random number list. Each patient was given one envelope by a staff nurse who was unaware of the study.
Bias due to deviations from intended interventions	Low risk	Double-Blind. The patients were blinded to the three study. The observers were blinded to the three study groups.
Bias due to missing outcome data	Some concerns	There are likely errors that remain in pain scores and the information was insufficient to make a judgment.
Bias in measurement of the outcome	Low risk	The method of measuring was appropriate.
Bias in selection of the reported result	Low risk	Pre-specified outcomes reported.
Overall bias	Some concerns	The overall risk of bias was classified as some concerns.

5 Mahran 2015
RoB 2 tool

Bias	Author's judgement	Support for judgement
Bias arising from the randomization process	Low risk	Using a computer-generated random number assignment. Allocations were concealed in sequentially numbered sealed opaque envelopes.
Bias due to deviations from intended interventions	Low risk	Both patients and postoperative assessors blinded to intraoperative management. An anesthesiologist not related to the management of the patient or study prepared the drugs of the study according to randomization.
Bias due to missing outcome data	Low risk	Analyses included almost all subjects. The exclusion of patients did not affect the study result.

Bias in measurement of the outcome	Low risk	The method of measuring was appropriate.
Bias in selection of the reported result	Low risk	Pre-specified outcomes reported.
Overall bias	Low risk	The overall risk of bias was classified as low risk.

6. Mansor 2015
RoB 2 tool

Bias	Author's judgement	Support for judgement
Bias arising from the randomization process	Low risk	Using computer-generated randomized numbers.
Bias due to deviations from intended interventions	Low risk	Patients were instructed to close their eyes before given the test drug to swallow.
Bias due to missing outcome data	Low risk	Analyses included almost all subjects. The exclusion of patients did not affect the study result.
Bias in measurement of the outcome	Low risk	The method of measuring was appropriate.
Bias in selection of the reported result	Low risk	Pre-specified outcomes reported.
Overall bias	Low risk	The overall risk of bias was classified as low risk.

7. Mohamed 2016
RoB 2 tool

Bias	Author's judgement	Support for judgement
Bias arising from the randomization process	Low risk	Randomization was done using lottery method.

Bias due to deviations from intended interventions	Low risk	Anesthesiologists and patients were blinded to the groups. Pregabalin was given orally by a staff nurse who was not included in the study.
Bias due to missing outcome data	Low risk	Analyses included almost all subjects. The exclusion of patients did not affect the study result.
Bias in measurement of the outcome	Low risk	The method of measuring was appropriate.
Bias in selection of the reported result	Low risk	Pre-specified outcomes reported.
Overall bias	Low risk	The overall risk of bias was classified as low risk.

8. Patel 2016
RoB 2 tool

Bias	Author's judgement	Support for judgement
Bias arising from the randomization process	Some concerns	Ninety patients were randomly allocated in 3 Groups. Did not mention randomization method.
Bias due to deviations from intended interventions	Some concerns	The patients were randomized in a double-blind manner. Did not mention masking method.
Bias due to missing outcome data	Low risk	Analyses included almost all subjects. The exclusion of patients did not affect the study result.
Bias in measurement of the outcome	Low risk	The method of measuring was appropriate.
Bias in selection of the reported result	Low risk	Pre-specified outcomes reported.
Overall bias	Some concerns	The overall risk of bias was classified as some concerns.

9. SK 2016

RoB 2 tool

Bias	Author’s judgement	Support for judgement
Bias arising from the randomization process	Low risk	Using computer-generated randomized numbers.
Bias due to deviations from intended interventions	Low risk	The patients were randomized in a double-blind manner. Anesthesia by a staff nurse who was not involved in the study. Both these outcomes were assessed by an independent anesthesiologist blinded to group allocation.
Bias due to missing outcome data	Low risk	Analyses included almost all subjects. The exclusion of patients did not affect the study result.
Bias in measurement of the outcome	Low risk	The method of measuring was appropriate.
Bias in selection of the reported result	Low risk	Pre-specified outcomes reported.
Overall bias	Low risk	The overall risk of bias was classified as low risk.

10. Pushkarna 2022
RoB 2 tool

Bias	Author’s judgement	Support for judgement
Bias arising from the randomization process	Low risk	Randomization was carried out using random numbers. The coded slips which were then made were put in a sealed envelope. The hospital pharmacy prepared capsules identical in size, shape and colour.
Bias due to deviations from intended interventions	Low risk	The patients remained blinded to the study drug. The assessors remained blinded to the study drug.
Bias due to missing outcome data	Low risk	Analyses included almost all subjects. The exclusion of patients did not affect the study result.

Bias in measurement of the outcome	Low risk	The method of measuring was appropriate.
Bias in selection of the reported result	Low risk	Pre-specified outcomes reported.
Overall bias	Low risk	The overall risk of bias was classified as low risk.

11. Salah 2018
RoB 2 tool

Bias	Author's judgement	Support for judgement
Bias arising from the randomization process	Some concerns	The patients are randomly allocated into two groups. Did not mention randomization method. This study was randomized by sealed opaque envelope.
Bias due to deviations from intended interventions	Low risk	The patients were not informed about the administered medications. The residents were not informed about the administered medications.
Bias due to missing outcome data	Low risk	Analyses included almost all subjects. The exclusion of patients did not affect the study result.
Bias in measurement of the outcome	Low risk	The method of measuring was appropriate.
Bias in selection of the reported result	Low risk	Pre-specified outcomes reported.
Overall bias	Some concerns	The overall risk of bias was classified as some concerns.

12. Zhang 2012
RoB 2 tool

Bias	Author's judgement	Support for judgement
Bias arising from the randomization process	Some concerns	The patients are randomly allocated into two groups. Did not mention randomization method.

Bias due to deviations from intended interventions	Some concerns	The patients were randomized in a double-blind manner. Did not mention masking method.
Bias due to missing outcome data	Low risk	Analyses included almost all subjects. The exclusion of patients did not affect the study result.
Bias in measurement of the outcome	Low risk	The method of measuring was appropriate.
Bias in selection of the reported result	Low risk	Pre-specified outcomes reported.
Overall bias	Some concerns	The overall risk of bias was classified as some concerns.

13. Zhang 2016
RoB 2 tool

Bias	Author's judgement	Support for judgement
Bias arising from the randomization process	Some concerns	The patients are randomly allocated into two groups. Did not mention randomization method.
Bias due to deviations from intended interventions	Some concerns	The patients were randomized in a double-blind manner. Did not mention masking method.
Bias due to missing outcome data	Low risk	Analyses included almost all subjects. The exclusion of patients did not affect the study result.
Bias in measurement of the outcome	Low risk	The method of measuring was appropriate.
Bias in selection of the reported result	Low risk	Pre-specified outcomes reported.
Overall bias	Some concerns	The overall risk of bias was classified as some concerns.

Table S2: GRADE quality of evidence summary table

Patient or population: Patients who underwent cancer-related surgery
Settings: RCTs.
Intervention: Pregabalin was administered preoperatively.
Comparison: The control interventions were placebo or no treatment.

**Outcomes**	**Illustrative comparative risks* (97.5% CI or 95% CI)**		**Relative effect (97.5% CI or 95% CI)**	**No of Participants (studies)**	**Quality of the evidence (GRADE)**	**Comments**
	Assumed risk **Control**	Corresponding risk **Outcomes**				
**The primary outcomes**						
**Resting pain scores at 24 hours postoperatively (cm)**		The mean resting pain scores at 24 hours postoperatively (cm) in the intervention groups was **0.45 lower** (0.66 to 0.24 lower)	-0.45 (-0.68 to -0.21)	580 (9 studies)	⊕⊕○○ **low**[1,2]	Pregabalin likely results in **a certain decrease** in resting pain scores at 24 hours postoperatively.
**Dynamic pain scores at 24 hours postoperatively (cm)**		The mean dynamic pain scores at 24 hours postoperatively (cm) in the intervention groups was **0.31 lower** (0.77 lower to 0.15 higher)	-0.31 (-0.83 to 0.22)	490 (7 studies)	⊕⊕○○ **low**[1,2]	Pregabalin likely results in **no difference** in dynamic pain scores at 24 hours postoperatively.
**The secondary outcomes**						

**Resting pain scores at 1 hour postoperatively (cm)**		The mean resting pain scores at 1 hour postoperatively (cm) in the intervention groups was **1.56 lower** (3.63 lower to 0.52 higher)	-1.56 (-3.63 to 0.52)	165 (2 studies)	⊕⊕⊕○ **moderate**[2]	Pregabalin likely results in **no difference** in resting pain scores at 1 hours postoperatively.
**Resting pain scores at 2 hours postoperatively (cm)**		The mean resting pain scores at 2 hours postoperatively (cm) in the intervention groups was **1.53 lower** (2.3 to 0.77 lower)	-1.53 (-2.30 to -0.77)	510 (7 studies)	⊕⊕⊕○ **moderate**[2]	Pregabalin likely results in **a certain decrease** in resting pain scores at 2 hours postoperatively.
**Resting pain scores at 4 hours postoperatively (cm)**		The mean resting pain scores at 4 hours postoperatively (cm) in the intervention groups was **0.53 lower** (0.98 to 0.08 lower)	-0.53 (-0.98 to -0.08)	465 (7 studies)	⊕⊕○○ **low**[2,4]	Pregabalin likely results in **a certain decrease** in resting pain scores at 4 hours postoperatively.
**Resting pain scores at 6 hours postoperatively (cm)**		The mean resting pain scores at 6 hours postoperatively (cm) in the intervention groups was **0.87 lower** (1.58 to 0.16 lower)	-0.87 (-1.58 to -0.16)	429 (7 studies)	⊕⊕⊕○ **moderate**[2,3]	Pregabalin likely results in **a certain decrease** in resting pain scores at 6 hours postoperatively.
**Resting pain scores at 8 hours postoperatively (cm)**		The mean resting pain scores at 8 hours postoperatively (cm) in the intervention groups was **0.64 lower** (0.96 to 0.32 lower)	-0.64 (-0.96 to -0.32)	311 (4 studies)	⊕⊕○○ **low**[1,2]	Pregabalin likely results in **a certain decrease** in resting pain scores at 8 hours postoperatively.

**Resting pain scores at 12 hours postoperatively (cm)**		The mean resting pain scores at 12 hours postoperatively (cm) in the intervention groups was **0.59 lower** (1.06 to 0.12 lower)	-0.59 (-1.06 to -0.12)	500 (8 studies)	⊕⊕○○ **low**[1,2]	Pregabalin likely results in **a certain decrease** in resting pain scores at 12 hours postoperatively.
**Resting pain scores at 16 hours postoperatively (cm)**		The mean resting pain scores at 16 hours postoperatively (cm) in the intervention groups was **1.07 lower** (1.88 to 0.25 lower)	-1.07 (-1.88 to -0.25)	246 (3 studies)	⊕⊕○○ **low**[1,2]	Pregabalin likely results in **a certain decrease** in resting pain scores at 16 hours postoperatively.
**Resting pain scores at 20 hours postoperatively (cm)**		The mean resting pain scores at 20 hours postoperatively (cm) in the intervention groups was **0.61 lower** (1.18 to 0.05 lower)	-0.61 (-1.18 to -0.05)	135 (2 studies)	⊕⊕○○ **low**[1,2]	Pregabalin likely results in **a certain decrease** in resting pain scores at 20 hours postoperatively.
**Resting pain scores at 48 hours postoperatively (cm)**		The mean resting pain scores at 48 hours postoperatively (cm) in the intervention groups was **0.13 lower** (0.42 lower to 0.15 higher)	-0.13 (-0.42 to 0.15)	90 (2 studies)	⊕⊕⊕○ **moderate**[1]	Pregabalin likely results in **no difference** in resting pain scores at 48 hours postoperatively.
**Dynamic pain scores at 2 hours postoperatively (cm)**		The mean dynamic pain scores at 2 hours postoperatively (cm) in the intervention groups was **1.16 lower** (2.22 to 0.11 lower)	-1.16 (-2.22 to -0.11)	400 (5 studies)	⊕⊕⊕○ **moderate**[2]	Pregabalin likely results in **a certain decrease** in dynamic pain scores at 2 hours postoperatively.

**Dynamic pain scores at 4 hours postoperatively (cm)**		The mean dynamic pain scores at 4 hours postoperatively (cm) in the intervention groups was **0.53 lower** (0.97 to 0.1 lower)	-0.53 (-0.97 to -0.10)	280 (4 studies)	⊕⊕○○ **low**[1,2]	Pregabalin likely results in **a certain decrease** in dynamic pain scores at 4 hours postoperatively.
**Dynamic pain scores at 6 hours postoperatively (cm)**		The mean dynamic pain scores at 6 hours postoperatively (cm) in the intervention groups was **1.03 lower** (1.83 to 0.23 lower)	-1.03 (-1.83 to -0.23)	319 (5 studies)	⊕⊕⊕○ **moderate**[2]	Pregabalin likely results in **a certain decrease** in dynamic pain scores at 6 hours postoperatively.
**Dynamic pain scores at 8 hours postoperatively (cm)**		The mean dynamic pain scores at 8 hours postoperatively (cm) in the intervention groups was **0.36 lower** (0.78 lower to 0.06 higher)	-0.36 (-0.78 to 0.06)	171 (2 studies)	⊕⊕○○ **low**[1,2]	Pregabalin likely results in **no difference** in dynamic pain scores at 8 hours postoperatively.
**Dynamic pain scores at 12 hours postoperatively (cm)**		The mean dynamic pain scores at 12 hours postoperatively (cm) in the intervention groups was **0.85 lower** (1.49 to 0.21 lower)	-0.85 (-1.49 to -0.21)	330 (5 studies)	⊕⊕○○ **low**[1,2]	Pregabalin likely results in **a certain decrease** in dynamic pain scores at 12 hours postoperatively.
**Dynamic pain scores at 16 hours postoperatively (cm)**		The mean dynamic pain scores at 16 hours postoperatively (cm) in the intervention groups was **0.26 lower** (0.54 lower to 0.02 higher)	-0.26 (-0.54 to 0.02)	171 (2 studies)	⊕⊕⊕○ **moderate**[1]	Pregabalin likely results in **no difference** in dynamic pain scores at 16 hours postoperatively.

**Dynamic pain scores at 48 hours postoperatively (cm)**		The mean dynamic pain scores at 48 hours postoperatively (cm) in the intervention groups was **0.31 lower** (1.17 lower to 0.54 higher)	-0.31 (-1.17 to 0.54)	90 (2 studies)	⊕⊕◯◯ **low**[1,2]	Pregabalin likely results in **no difference** in dynamic pain scores at 48 hours postoperatively.
**Cumulative morphine equivalent consumption within 12 hours (mg)**		The mean cumulative morphine equivalent consumption within 12 hours (mg) in the intervention groups was **1.77 lower** (6.77 lower to 3.24 higher)	-1.77 (-6.77 to 3.24)	110 (2 studies)	⊕⊕⊕◯ **moderate**[2]	Pregabalin likely results in **no difference** in cumulative morphine equivalent consumption within 12 hours.
**Cumulative morphine equivalent consumption within 24 hours (mg)**		The mean cumulative morphine equivalent consumption within 24 hours (mg) in the intervention groups was **7.45 lower** (9.3 to 5.6 lower)	-7.45 (-9.30 to -5.60)	646 (10 studies)	⊕◯◯◯ **very low**[1,2,4]	Pregabalin likely results in **a certain decrease** in cumulative morphine equivalent consumption within 24 hours.
**Cumulative morphine equivalent consumption within 48 hours (mg)**		The mean cumulative morphine equivalent consumption within 48 hours (mg) in the intervention groups was **29.93 lower** (81.99 lower to 22.13 higher)	-29.93 (-81.99 to 22.13)	90 (2 studies)	⊕◯◯◯ **very low**[1,2,4]	Pregabalin likely results in **no difference** in cumulative morphine equivalent consumption within 48 hours.
**Time to first analgesic request (hours)**		The mean time to first analgesic request (hours) in the intervention groups was **2.28 higher** (0.79 to 3.77 higher)	2.28 (0.79 to 3.77)	255 (4 studies)	⊕◯◯◯ **very low**[1,2,3,4]	Pregabalin likely results in **a certain increase** in time to first analgesic request.

**Hemodynamic parameters**						
**Heart rate at 2 hours (beat/min)**		The mean heart rate at 2 hours (beat/min) in the intervention groups was **2.81 lower** (7.71 lower to 2.08 higher)	-2.81 (-7.71 to 2.08)	135 (2 studies)	⊕⊕⊕⊕ **high**	Pregabalin likely results in **no difference** in heart rate at 2 hours.
**Heart rate at 6 hours (beat/min)**		The mean heart rate at 6 hours (beat/min) in the intervention groups was **2.53** lower (7.59 lower to 2.53 higher)	-2.53 (-7.59 to 2.53)	135 (2 studies)	⊕⊕⊕⊕ **high**	Pregabalin likely results in **no difference** in heart rate at 6 hours.
**Heart rate at 12 hours (beat/min)**		The mean heart rate at 12 hours (beat/min) in the intervention groups was **3.83 lower** (8.59 lower to 0.92 higher)	-3.83 (-8.59 to 0.92)	135 (2 studies)	⊕⊕⊕⊕ **high**	Pregabalin likely results in **no difference** in heart rate at 12 hours.
**Heart rate at 24 hours (beat/min)**		The mean heart rate at 24 hours (beat/min) in the intervention groups was **2.73 lower** (7.44 lower to 1.98 higher)	-2.73 (-7.44 to 1.98)	135 (2 studies)	⊕⊕⊕⊕ **high**	Pregabalin likely results in **no difference** in heart rate at 24 hours.
**SBP at 2 hours (mm/Hg)**		The mean SBP at 2 hours (mm/hg) in the intervention groups was	-9.14 (-19.88 to 1.59)	135 (2 studies)	⊕⊕⊕○ **moderate**[2]	Pregabalin likely results in **no difference** in SBP at 2 hours.

		**9.14 lower**<br>(19.88 lower to 1.59 higher)				
**SBP at 6 hours (mm/Hg)**		The mean SBP at 6 hours (mm/hg) in the intervention groups was **1.79 lower**<br>(6.31 lower to 2.72 higher)	-1.79 (-6.31 to 2.72)	135<br>(2 studies)	⊕⊕⊕⊕<br>**high**	Pregabalin likely results in **no difference** in SBP at 6 hours.
**SBP at 12 hours (mm/Hg)**		The mean SBP at 12 hours (mm/hg) in the intervention groups was **3.31 higher**<br>(5.59 lower to 12.22 higher)	3.31 (-5.59 to 12.22)	135<br>(2 studies)	⊕⊕⊕○<br>**moderate**[2]	Pregabalin likely results in **no difference** in SBP at 12 hours.
**SBP at 24 hours (mm/Hg)**		The mean SBP at 24 hours (mm/hg) in the intervention groups was **0.25 higher**<br>(4.59 lower to 5.08 higher)	0.25 (-4.59 to 5.08)	135<br>(2 studies)	⊕⊕⊕⊕<br>**high**	Pregabalin likely results in **no difference** in SBP at 24 hours.
**DBP at 2 hours (mm/Hg)**		The mean DBP at 2 hours (mm/hg) in the intervention groups was **0.13 lower**<br>(4.06 lower to 3.81 higher)	-0.13 (-4.06 to 3.81)	135<br>(2 studies)	⊕⊕⊕⊕<br>**high**	Pregabalin likely results in **no difference** in DBP at 2 hours.
**DBP at 6 hours (mm/Hg)**		The mean DBP at 6 hours (mm/hg) in the intervention groups was **2.26 lower**<br>(5.45 lower to 0.93 higher)	-2.26 (-5.45 to 0.93)	135<br>(2 studies)	⊕⊕⊕⊕<br>**high**	Pregabalin likely results in **no difference** in DBP at 6 hours.
**DBP at 12 hours (mm/Hg)**		The mean DBP at 12 hours (mm/hg) in the intervention groups was	1.99 (-8.60 to 12.58)	135<br>(2 studies)	⊕⊕⊕○<br>**moderate**[2]	Pregabalin likely results in **no difference** in DBP at 12 hours.

		**1.99 higher**<br>(8.6 lower to 12.58 higher)				
**DBP at 24 hours (mm/Hg)**		The mean DBP at 24 hours (mm/hg) in the intervention groups was<br>**1.55 lower**<br>(6.59 lower to 3.5 higher)	-1.55 (-6.59 to 3.50)	135<br>(2 studies)	⊕⊕⊕○<br>**moderate**[2]	Pregabalin likely results in **no difference** in DBP at 24 hours.
**The safety outcomes**						
**Dizziness**	**Study population**		**RR 2.81**<br>(1.75 to 4.53)	490<br>(7 studies)	⊕⊕⊕○<br>**moderate**[1]	Pregabalin likely results in **a certain increase** in incidence of dizziness.
	**91 per 1000**	**257 per 1000**<br>(160 to 414)				
	**Moderate**					
	**33 per 1000**	**93 per 1000**<br>(58 to 149)				
**Visual disturbance**	**Study population**		**RR 3.04**<br>(1.37 to 6.73)	415<br>(6 studies)	⊕⊕⊕○<br>**moderate**[1]	Pregabalin likely results in **a certain increase** in incidence of visual disturbance.
	**38 per 1000**	**115 per 1000**<br>(52 to 254)				
	**Moderate**					
	**0 per 1000**	**0 per 1000**<br>(0 to 0)				
**Pruritus**	**Study population**		**RR 0.14**<br>(0.02 to 1.02)	220<br>(4 studies)	⊕⊕⊕○<br>**moderate**[1]	Pregabalin likely results in **no difference** in incidence of pruritus.
	**43 per 1000**	**6 per 1000**<br>(1 to 44)				
	**Moderate**					

	**17 per 1000**	**2 per 1000** (0 to 17)				
**Headache**	**Study population**		**RR 1.71** (0.88 to 3.31)	295 (4 studies)	⊕⊕⊕⊕ **high**	Pregabalin likely results in **no difference** in incidence of headache.
	**111 per 1000**	**190 per 1000** (98 to 368)				
	**Moderate**					
	**50 per 1000**	**86 per 1000** (44 to 165)				
**Sedation score at 12 hours**		The mean sedation score at 12 hours in the intervention groups was **0.35 higher** (0.15 to 0.55 higher)	0.35 (0.15 to 0.55)	230 (3 studies)	⊕⊕⊕⊕ **high**	Pregabalin likely results in **a certain increase** in sedation score at 12 hours.
**Sedation score at 24 hours**		The mean sedation score at 24 hours in the intervention groups was **0.5 higher** (0.15 to 0.86 higher)	0.50 (0.15 to 0.86)	261 (3 studies)	⊕⊕⊕○ **moderate**[2]	Pregabalin likely results in **a certain increase** in sedation score at 24 hours.
**PONV**	**Study population**		**RR 0.59** (0.39 to 0.87)	670 (10 studies)	⊕⊕○○ **low**[1,2]	Pregabalin likely results in **a certain decrease** in incidence of PONV.
	**372 per 1000**	**219 per 1000** (145 to 324)				
	**Moderate**					
	**350 per 1000**	**206 per 1000** (136 to 305)				

*The basis for the **assumed risk** (e.g., the median control group risk across studies) is provided in footnotes. The **corresponding risk** (and its 95% or 99% confidence interval) is based on the assumed risk in the comparison group and the **relative effect** of the intervention (and its 95% CI or 99%

CI).

Abbreviation: GRADE: Grades of Recommendation, Assessment, Development, and Evaluation; **RCTs**: Randomized controlled trials; **CI:** Confidence interval; **RR:** Risk ratio; **N:** Number; **cm**: centimeter; **mg**: milligrams; **SBP**: systolic blood pressure; **DBP**: diastolic blood pressure; **PONV**: Postoperative Nausea and Vomiting.

GRADE Working Group grades of evidence

High quality: Further research is very unlikely to change our confidence in the estimate of effect.

Moderate quality: Further research is likely to have an important impact on our confidence in the estimate of effect and may change the estimate.

Low quality: Further research is very likely to have an important impact on our confidence in the estimate of effect and is likely to change the estimate.

Very low quality: We are very uncertain about the estimate.

[1] High risk of bias

[2] High heterogeneity

[3] Some evidence of imprecision such as wide confidence interval due to small sample size

[4] Publication bias

Table S3: Meta-regression analysis for primary outcomes

Subgroup	N of studies	$R^{2\,*}$	Wald Chi^2	*p*
**Resting pain scores at 24 hours postoperatively (cm)**				
Types of cancer	9	0	0.14	0.712
Dose of pregabalin	13	0	0.06	0.800
Types of surgery	9	0	0.20	0.654
Methods of postoperative rescue analgesia	9	0.23	3.66	0.056
Surgical site	9	0	0.13	0.717
Postoperative multimodal analgesia	9	0.22	1.63	0.202
**Dynamic pain scores at 24 hours postoperatively (cm)**				
Types of cancer	7	0	0.04	0.847
Dose of pregabalin	10	0	0.29	0.587
Types of surgery	7	0	0.07	0.890
Methods of postoperative rescue analgesia	7	0.54	3.95	0.047
Surgical site	7	0	0.04	0.846
Postoperative multimodal analgesia	7	0.437	1.12	0.290

[*] An R^2 value (coefficient of determination) was calculated to help quantify the extent of a covariate explained the variation in data. An R^2=1 denoted that the covariate explained all the variability, while an R^2=0 denoted that the covariate did not explain any of the variability.

Abbreviation: **N**: Number; **N/A**: Not applicable; **cm**: centimeter.

Table S4A: Subgroup analyses for primary and safety outcomes

Subgroup	Subgroup category	Included studies	N of studies	WMD or RR (95% CI or 97.5% CI)	Model	*P*	I^2 test (%)
**The primary outcomes**							
**Dynamic pain scores at 24 hours postoperatively (cm)**							
Methods of postoperative rescue analgesia	PCA	Earsakul 2017, Ghoneim 2013, Hetta 2016, Mahran 2015	4	-0.14 (-0.42, 0.13)	Radom	0.39	0
	IV	Mansor 2015	1	-0.01 (-0.20, 0.18)	Radom	N/A	N/A
	IM	Patel 2016, Zhang 2016	2	-0.87 (-1.42, -0.33)	Radom	0.01	86.11
**The safety outcomes**							
**Dizziness**							
Dose of pregabalin	Low dose	Ghoneim 2013, Hetta 2016 a, Hetta 2016 b, Mahran 2015, Mansor 2015, Patel 2016 a, Zhang 2012	7	2.20 (1.38, 3.48)	Fixed	<0.001	0
	High dose	Hetta 2016 c, Mohamed 2016 a, Mohamed 2016 b, Patel 2016 b	4	9.25 (3.22, 26.54)	Fixed	<0.001	9
**Visual disturbance**							

Dose of pregabalin	Low dose	Ghoneim 2013, Hetta 2016 a, Hetta 2016 b, Mahran 2015, Mansor 2015, Patel 2016 a	6	2.13 (1.02, 4.47)	Fixed	0.05	0
	High dose	Hetta 2016 c, Mohamed 2016 a, Mohamed 2016 b, Patel 2016 b	4	7.25 (2.75, 19.07)	Fixed	<0.001	0
**Pruritus**							
Dose of pregabalin	Low dose	Ghoneim 2013, Lamsal 2019 a, Lamsal 2019 b, Pushkarna 2022	4	0.33 (0.01, 7.87)	Fixed	N/A	N/A
	High dose	Mohamed 2016 a, Mohamed 2016 b	2	0.14 (0.02, 1.10)	Fixed	0.06	0
**Headache**							
Dose of pregabalin	Low dose	Hetta 2016 a, Hetta 2016 b, Mansor 2015, Patel 2016 a	4	1.33 (0.70, 2.53)	Fixed	0.38	0
	High dose	Hetta 2016 c, Mohamed 2016 a, Patel 2016 b, Mohamed 2016 b	4	5.11 (1.70, 15.36)	Fixed	0.004	0
**Sedation score at 12 hours**							
Dose of pregabalin	Low dose	Patel 2016 a, SK 2016	2	0.35 (0.15, 0.55)	Fixed	<0.001	N/A
	High dose	Patel 2016 b, Salah 2018	2	N/A	Fixed	N/A	N/A

**Sedation score at 24 hours**							
Dose of pregabalin	Low dose	Hetta 2016 a, Hetta 2016 b, Patel 2016 a	3	0.14 (-0.09, 0.37)	Fixed	0.23	0
	High dose	Hetta 2016 c, Patel 2016 b, Salah 2018	3	0.76 (0.54, 0.97)	Fixed	<0.001	0
**POVN**							
Dose of pregabalin	Low dose	Ghoneim 2013, Hetta 2016 a, Hetta 2016 b, Lamsal 2019 a, Lamsal 2019 b, Mahran 2015, Mansor 2015, Patel 2016 a, SK 2016, Pushkarna 2022	10	0.70 (0.55, 0.90)	Radom	0.005	24
	High dose	Hetta 2016 c, Mohamed 2016 a, Mohamed 2016 b, Patel 2016 b, Salah 2018	5	0.32 (0.19, 0.52)	Radom	<0.001	0

Abbreviation: N: Number; **WMD**: Weighted mean difference; **RR**: Risk ratio; **CI**: Confidence interval; $\mathbf{I^2}$: I-square; **N/A**: Not applicable; **PCA**: Patient controlled analgesia; **IV**: Intravenous injection; **IM**: Intramuscular injection; **PONV:** Postoperative Nausea and Vomiting.

Table S4B: Predefined sensitivity analyses for primary outcomes

Deleted Studies	WMD (97.5% CI)	Model	*P*	I^2 test (%)	N of studies
**Resting pain scores at 24 hours postoperatively (cm)**					
Mansor 2015	-0.49 (-0.76, -0.22)	Radom	<0.001	54.69	8
**Dynamic pain scores at 24 hours postoperatively (cm)**					
Mansor 2015	-0.38 (-0.91, 0.15)	Random	0.11	88.71	6

Abbreviation: WMD: Weighted mean difference; **RR**: Risk ratio; **CI**: Confidence interval; $\mathbf{I^2}$: I-square; **N**: Number.

Figure S1: Risk of bias summary

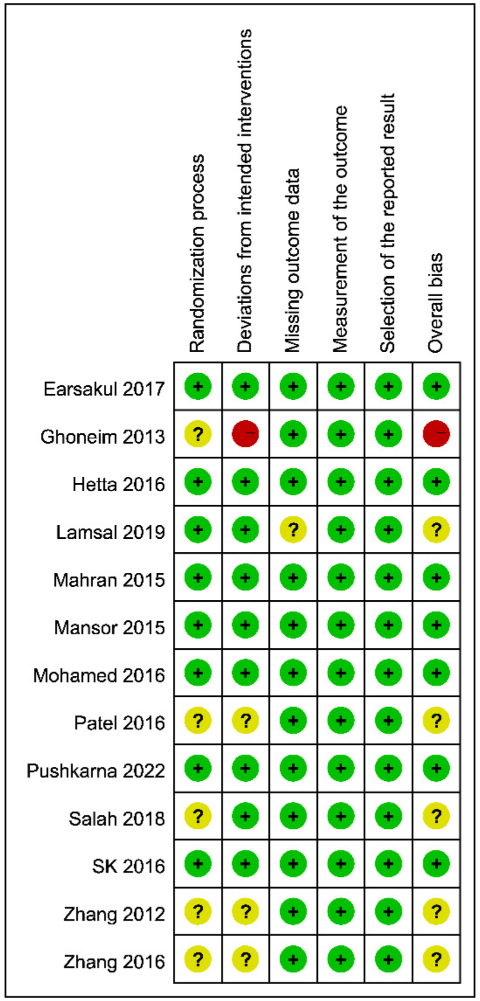

Supplement: Supplementary file 1 [file medicina-59-00280-s001.zip › medicina-2128816-supplementary.pdf]
